# Anti-CD38 targeted nanotrojan horses stimulated by acoustic waves as therapeutic nanotools selectively against Burkitt’s lymphoma cells

**DOI:** 10.1186/s11671-024-03976-z

**Published:** 2024-02-14

**Authors:** Veronica Vighetto, Marzia Conte, Giada Rosso, Marco Carofiglio, Federica Sidoti Abate, Luisa Racca, Giulia Mesiano, Valentina Cauda

**Affiliations:** 1 Department of Applied Science and Technology, Politecnico di Torino, 10129 Turin, Italy; 2 Present Address: Department of Biochemistry and Molecular Pharmacology, Istituto di Ricerche Farmacologine Mario Negri, IRCCS, 20156 Milan, Italy; 3 Present Address: Department of Translational Medicine, University of Piemonte Orientale, 28100 Novara, Italy

**Keywords:** Ultrasounds, Monoclonal antibody fragments, Zinc oxide, Biomimetic nanoparticles, Targeted therapy, Stimuli-responsive nanoparticles

## Abstract

The horizon of nanomedicine research is moving toward the design of therapeutic tools able to be completely safe per se, and simultaneously be capable of becoming toxic when externally activated by stimuli of different nature. Among all the stimuli, ultrasounds come to the fore as an innovative approach to produce cytotoxicity on demand in presence of NPs, without invasiveness, with high biosafety and low cost. In this context, zinc oxide nanoparticles (NPs) are among the most promising metal oxide materials for theranostic application due to their optical and semi-conductor properties, high surface reactivity, and their response to ultrasound irradiation. Here, ZnO nanocrystals constitute the stimuli-responsive core with a customized biomimicking lipidic shielding, resembling the composition of natural extracellular vesicles. This core–shell hybrid structure provides high bio- and hemocompatibility towards healthy cells and is here proofed for the treatment of Burkitt’s Lymphoma. This is a very common haematological tumor, typically found in children, for which consolidated therapies are so far the combination of chemo-therapy drugs and targeted immunotherapy. In this work, the proposed safe-by-design antiCD38-targeted hybrid nanosystem exhibits an efficient selectivity toward cancerous cells, and an on-demand activation, leading to a significant killing efficacy due to the synergistic interaction between US and targeted hybrid NPs. Interestingly, this innovative treatment does not significantly affect healthy B lymphocytes nor a negative control cancer cell line, a CD38- acute myeloid leukemia, being thus highly specific and targeted. Different characterization and analyses confirmed indeed the effective formation of targeted hybrid ZnO NPs, their cellular internalization and the damages produced in Burkitt’s Lymphoma cells only with respect to the other cell lines. The presented work holds promises for future clinical applications, as well as translation to other tumor types.

**Graphical abstract:**

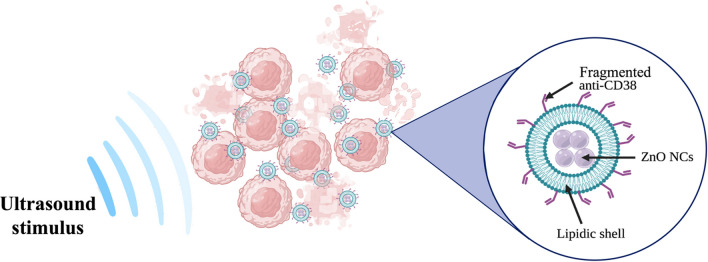

**Supplementary Information:**

The online version contains supplementary material available at 10.1186/s11671-024-03976-z.

## Introduction

Nanomedicine and nanotheranostic perspectives are nowadays oriented toward the design of therapeutic tools which can be per se completely safe and become toxic only when externally activated on demand by a stimulus. In this perspective, inorganic nanoparticles (NPs) are widely studied for stimuli-responsive activation to perform therapy, specifically against cancer [1]. One of the most studied external stimuli is light, and different light sensitive nanomaterials have been developed, especially for diagnostic purposes [2]. The combination of nanoparticles and light is also proposed in nanomedicine research as therapy, i.e. photodynamic therapy (PDT) [1]. The efficacy of this treatment against cancer relies on the generation of Reactive Oxygen Species (ROS) when light react with the nanoparticles’ surface, due to the role that ROS play in regulating tumor vasculature, in inducing inflammatory responses and in the activation of antitumor immune system-specific reactions [1, 3]. However, this external way to activate NPs present some limitations, such as the poor penetration of light, which limits the applicability of the treatment up to few millimeters deep in the skin, and the low efficacy of PDT when the interested tissue is affected by hypoxia, as it occurs in most cases of solid tumors [4]. Other limitations, including dark toxicity, low tumor tissue selectivity, low photostability and poor biocompatibility of the photosensitizers restrict the clinical use of PDT in cancer therapy. Magnetic and microwave (MW) stimuli were also deeply studied in combination with NPs, to achieve overheating of a specific tissue obtaining tumor tissue thermoablation [5]. Superparamagnetic nanoparticles are able to convert the electromagnetic energy provided by an alternate magnetic field into heat, causing local temperature increase in cells close to NPs [6]. However, this approach presents different disadvantages, which limit the applicability of this technique. The efficacy of activated magnetic NPs as therapeutic agents is actually affected by the occurrence of undesired cell necrosis which can lead to cancer metastasis and inflammatory disease [7]. Otherwise, NPs can be stimulated with MW to produce heat, however, this approach requires the use of a needle that must be inserted into the patient, resulting in bleeding, especially in case of multiple treatments, or infections [1, 5].

Among all the above-mentioned stimuli and some additional ones—like radiofrequency or electric field triggering—investigated in the literature to activate NPs, ultrasounds (US) come to the fore as an innovative approach to produce cytotoxicity on demand in solid tumors in a site-directed manner when in presence of NPs [8]. Non-invasiveness, high biosafety and low cost are the main characteristic of US and the foundation of their extensive use in standard clinical procedures [9]. The high tissue-penetrating capability of US [9] can be exploited in synergy with safe-by-design nanoparticles to achieve on demand and localized cytotoxic effects for the eradication of tumors. The exposure of NPs to US irradiation produces the enhanced occurrence of different phenomena, as photon emission [10], advantageous for imaging purposes, ROS generation [11, 12] and microjets formation [11, 13, 14] able to create physico-chemical damages to tumoral cells, leading to cells apoptosis or necrosis and constructing an effective theranostic tool. Recently Zinc oxide nanocrystals (ZnO NCs) has been proposed as effective sonosensitizing nanoparticles, able to produce dramatic effect when applied in combination with ultrasound [11] or shock waves [13] to different types of cancer cells [14, 15], yet producing ROS, zinc cation release and mechanical damages. Actually, zinc oxide nanoparticles are among the most promising materials for theranostic applications [14, 16] due to their wide band gap [17], suitable for optical imaging, their high surface reactivity, and their response to ultrasound irradiation [10, 11, 13, 18] leading to the above mentioned physico-chemical damages to cells. Furthermore, ZnO has been proven to be selectively toxic per se against cancer cells [16, 19] and more tolerated by healthy ones. It can also fully biodegrade into cytotoxic zinc cations due to the reduced pH of endosomal and lysosomal compartments, once internalized into cells, which is of course a further useful weapon when treating cancer cells in a targeted manner.

However, ZnO NPs and more in general inorganic NPs cannot be used as pristine material in contact with biological fluids, as they can easily aggregate, react or dissolve in biological environments; they can also be immunogenic and be non-specifically uptaken by various organs. Therefore, among the various strategies developed so far, one of the most promising one is to encase inorganic NPs in phospholipidic bilayers, thus providing a biomimetic shield [20, 21]. Such hybrid nanoparticle acquires therefore a plethora of qualities as higher biocompatibility, higher blood circulation, reduced clearance, reduced off target side effects [22, 23] with respect to the pristine inorganic NPs. Furthermore, inorganic NPs shielded by a phospholipid bilayer can be efficiently delivered towards different types of cancerous cells [24–26] exploiting either passive accumulation (EPR effect, Enhanced Permeability and Retention) or active targeting in conjugation to proper ligands [27]. This strategy should improve NPs stability and decrease off-target toxicity. Subsequently, the remote activation of such hybrid NPs can be exploited to selectively kill the target cell [28]. This approach can be generalized from bioimaging purposes up to therapeutic application with increased efficacy [29–31], revealing how their nanometric nature can be exploited for nanomedicine applications.

A very recent approach has proposed the design of lipid bilayer structures resembling the composition of natural extracellular vesicles (EVs) and mimicking their intrinsic homing capabilities towards recipient cells [32], but with a higher reproducibility, controlled size range, and the possibility to have massive production system [33, 34]. Furthermore, phospholipids can be effortlessly conjugated with PEG molecules, able to prolong the blood circulation time [35] of the final nanoparticles and consequently increasing the EPR effect. Phospholipids and PEG can be also conjugated with targeting peptides or antibodies to achieve site-selective targeting, and the resulting lipid bilayer shell offers the capability to contain moderate quantity of either hydrophobic or hydrophilic molecules.

In view of the above considerations, this paper proposes a targeted hybrid NPs composed by a core of zinc oxide nanocrystals (ZnO NCs) and a customized biomimicking lipidic shielding, composed by anionic and neutral phospholipids, cholesterol, and PEGylated lipids bioconjugated to antibody fragments. In particular, a fragmented anti-CD38 (the clinical-grade Daratumumab) is employed to drive the nanoconstruct against CD38 antigen overexpressed by Burkitt’s Lymphoma cells. In contrast, the selectivity towards a negative control cancer cell type, i.e. acute myeloid leukemia (HL60 cell line), not expressing CD38 antigen, does not occur.

After verifying the full bio and haemocompatibility of the hybrid core–shell ZnO nanocrystals coated by a lipidic biomimetic shell, towards both cancer cell lines and B lymphocytes as heathy counterpart, we demonstrate their on-demand therapeutic activation: the nanoparticles killing capability becomes effective only when externally activated by ultrasound irradiation and upon cell targeting, like nanosized Trojan horses. The proposed targeted hybrid system exhibits an efficient selectivity and cytotoxicity on-demand only toward cancerous Burkitt’s Lymphoma cells, without significantly affecting healthy B lymphocytes. We indeed show that our Trojan-nanohorse can be a powerful and selective tool against haematological cancer, opening the way to further explorations towards solid tumors in future.

## Methods

### Nanocrystals synthesis and functionalization

Zinc oxide nanocrystals were synthesized by a wet chemical process, exploiting oleic acid (Sigma-Aldrich) as stabilizing agent, as reported elsewhere[16, 36]. Briefly, zinc acetate dihydrate (526 mg, ACS Reagent, Sigma-Aldrich) was dissolved in 40 mL of ethanol and heated up to 70 °C. Bidistilled water (1 mL, from a Direct Q3 system, Millipore, Burlington) and oleic acid (140 µL) were added to the solution. Then, tetramethylammonium hydroxide (1.044 mg, TMAH, Sigma-Aldrich) previously dissolved in bidistilled water (1.052 mL) and ethanol (10 mL), was added to the zinc precursor solution to form the NCs. After 10 min, the NCs were collected and centrifuged to be resuspended in fresh ethanol. The procedure was repeated three times. ZnO NCs surface was then decorated with amino-propyl functional groups, adding 10 mol% of 3-aminopropyltrimethoxysilane (APTMS, Sigma-Aldrich) according to the literature [16, 17, 36]. This functionalization allows to increase the Z-potential towards positive values and thus to improve the colloidal stability of NCs. Furthermore it allows to covalently bind different moieties, such as fluorescent dyes on the NCs surfaces.

More in details, ZnO NCs were dispersed in ethanol at a concentration of 2.5 mg/mL and heated up to 70 °C in nitrogen atmosphere and refluxing conditions. APTMS was added to the solution and the reaction was carried on for 6 h at 70 °C. At the end of the procedure, NCs were collected and washed three times by a centrifugation and redispersion process. ZnO NCs were stored as ethanol colloidal suspensions, according to previous work [16, 37].

Dynamic Light Scattering (DLS) and Z-Potential measurements were carried out with Zetasizer Nano ZS90 (Malvern Instruments). The size of amino-propyl functionalized ZnO NCs was measured in bidistilled (bd) water at a concentration of 100 μg/mL. Z-Potential measurements were performed in bd water at a concentration of 100 μg/mL. Nanoparticles Tracking Analysis (NTA) measurements were executed on functionalized ZnO NCs with a NanoSight NS300 (Malvern Panalytical). To prepare the sample 6 μL of 1 mg/mL NCs solution were diluted up to 1 mL in bidistilled water. For each sample, three videos of 60 s of the samples fluxing in the instrument chamber were captured and analyzed with the NTA 3.4 software (Malvern Panalytical).

Functionalized ZnO nanocrystals were also characterized with Transmission Electron Microscopy (TEM). NCs were dispersed in water at a concentration of 50 μg/mL. Then, 10 μL of the solution were deposited onto a Lacey Carbon Support Film (300 mesh, Cu, Ted Pella Inc.) and let dry. The measurements were held with a Talos™ F200X G2 S(TEM) from Thermo Scientific at an operating voltage of 200 kV.

### Antibody reduction and lipid coupling

The reduced antibody was the FDA approved Anti-CD38 Daratumumab, with a concentration equal to 20 mg/mL. 100 μL of Ethylenediaminetetraacetic acid tetrasodium salt dihydrate (EDTA, Sigma-Aldrich) solution in phosphate saline buffer (PBS, Sigma-Aldrich) (10 mM) containing 200 μg of Anti-CD38 Daratumumab were prepared to be reduced by Tris(2-carboxyethyl)phosphine hydrochloride (TCEP). Previously, 200 μL of TCEP were centrifuged at 1000*g* for 30 s in a 1.5 mL microcentrifuge spin cup tube equipped with a filter (Thermo Scientific, 10 μm pore size); the solution filtered through the spin cup was discarded and the filter column containing the residual TCEP was placed in a new spin cup tube. The PBS/EDTA solution with Daratumumab was added to TCEP, delicately pipetting the solution in the gel. The tube was then placed for 24 h in a rotating wheel. The reduced sample was centrifuged at 1000*g* for 1 min and the filter was discarded.

To evaluate the concentration of antibody into the solution, the Bradford protein assay aimed at amino-acid quantification through a spectroscopic measurement was exploited. Bovin Serum Albumin (Sigma-Aldrich) dissolved in PBS was exploited to build a standard curve for protein evaluation. For the sample preparation, Daratumumab was diluted in PBS with a 1:16 ratio. In a 96 well plate (TC treated, Corning), 10 μL of both reference and sample solutions were added. Then, 200 μL of Coomassie brilliant blue G-250 dye (Bio-Rad) diluted 1:5 in bidistilled water was added to each well. The absorbance of the solutions at 590 nm was measured through a Multiskan GO microplate spectrophotometer (Thermo Fisher Scientific) and the values obtained were compared with the standard curve.

To verify the effectiveness of Daratumumab reduction, Sodium Dodecyl Sulphate–Polyacrylamide Gel Electrophoresis (SDS-PAGE) was performed. Criterion TGX stain free precast gel (45 μL wells, 4–15%, BioRad) was used and three sample were loaded for the test: a marker (precision plus protein dual color standard, BioRad), the whole anti-CD38 and the reduced anti-CD38. Sample containing the intact Daratumumab was prepared adding 2.25 μL of Daratumumab (1 mg/mL) to 24.75 μL of PBS and 9 μL of Laemmli Sample Buffer; 27 μL of reduced anti-CD38 was mixed with 9 μL of Laemmli Sample Buffer. Water sample was used to separate each sample lanes, and it was composed by 27 μL of bd water and 9 μL of Laemmli Sample Buffer. To complete samples preparation, they were heated at 95 °C for 10 min and loaded in precast gel, which was previously immersed in running buffer composed by 70 mL of 10 × Tris/Glicine SDS and 630 mL of bd water, inside the electrophoresis cell. The cell was closed and connected to the power supply (PowerPac™ Basic Power Supply) at constant voltage (200 V). At the end of the electrophoresis process, the gel was extracted from the cell, and underwent 3 washing steps with bd water, each lasting 5 min under orbital shaking at 50 rpm. Finally, the gel was stained by pouring 50 mL of Coomassie solution (Coomassie, BioSafe) on it, in continuous shaking at 50 rpm for 1 h. After removing the stain, the gel was washed 2 times, for 30 min each in orbital shaking at 50 rpm. After the last washing cycle, the gel was left overnight in the dark on orbital shaker. After one day a picture of the gel was acquired, to evaluate the reduction of anti-CD38 by electrophoresis, comparing molecular weights of whole anti-CD38 and fragmented anti-CD38 with the reference marker.

With the aim of creating a targeting liposome, the reduced antibody was conjugated with lipids. DSPE-PEG(2000)-Maleimide (1,2-distearoyl-sn-glycero-3-phosphoethanolamine-N-[maleimide(polyethylene glycol)-2000] (10 mg/mL), in a molar ratio with respect to reduced anti-CD38 equal to 3:1, was let evaporate in a glass vial. The lipid was rehydrated with Dimethyformamide (DMF) in a volume equal to the one of the reduced antibody solution. Then, the reduced anti-CD38 was added to the lipidic DMF solution and left at constant shaking at 250 rpm, at room temperature for 1 h. The conjugated anti-CD38 with DSPE-PEG(2000)-Maleimide was stored at -20 °C.

### Liposome formation and nanocrystals encapsulation

To enhance both the nanoparticles’ stability in aqueous media and their biocompatibility, a custom made lipidic coating was designed to be close to a simplified natural EV formulation. Briefly, a mixture of different commercially available lipids, including negatively-charged, amphiphatic and PEGylated lipids, and cholesterol was dried under vacuum overnight and then resuspended in a solution of ethanol and water. The solution was then used to coat the nanoparticles employing a solvent exchange technique previously reported in another work [38].

More in detail, a negatively charged lipid, DOPA (1,2-dioleoyl-sn-glycero-3-phosphate dissolved in chloroform, by Avanti Polar Lipids), a neutral lipid, DOPC (1,2-dioleoyl-sn-glycero-3-phosphocholine dissolved in chloroform, by Avanti Polar Lipids), a cholesterol solution in chloroform (Sigma Aldrich) and a PEGylated lipid, DSPE-PEG(2000)-Amine (1,2-distearoyl-sn-glycero-3-phosphoethanolamine-n-[amino(polyethylene glycol)-2000] dissolved in chloroform, Avanti Polar Lipids) were mixed in a molar ratio of 50/10/38.5/1.5 and dried overnight under vacuum. Afterwards, the dried mixture was resuspended in a solution made up of absolute ethanol and water, with a volume ratio of 40/60, to obtain a 3 mg/mL lipidic solution.

Resuspended lipids were hence added to a pellet of previously centrifuged nanoparticles, according to an optimized NCs/lipids weight ratio of 2/1. The mixture was sonicated with the help a sonication bath (Branson 3800 CPXH, Branson Ultrasonics Corporation) for 3 min at 59 kHz to better disperse the ZnO NCs into the lipidic solution. Thereafter, a volume of bidistilled water was added to allow the formation of the lipidic shell on the surface of the nanoparticles and a final 1 mg/mL concentration of lipid-coated NCs was achieved. A further sonication of 5 min was then carried out to obtain a homogeneous sample and to reduce the size of the newly formed lipidic shells.

A second lipid coating containing the reduced anti-CD38 was designed by adjusting some of the previously mentioned parameters: another PEGylated lipid, DSPE-PEG(2000)-Maleimide (1,2-distearoyl-sn-glycero-3-phosphoethanolamine-n-[maleimide(polyethylene glycol)-2000] dispersed in chloroform, Avanti Polar Lipids), previously conjugated to the reduced anti-CD38 as described above, was added with a molar ratio of 0.1% with respect to the total lipidic formulation and included in the 1.5% ratio of the DSPE-PEG(2000)-Amine lipid. The lipidic mixture containing the reduced antibody was dried overnight and the whole process was identically repeated to coat the ZnO NCs (ZnO-LipCD38).

DLS, Z-Potential and NTA measurements were performed on lipid-coated ZnO NCs (ZnO-Lip) as reported in the previous section, to characterize the nanoconstruct and underline the effectiveness of the encapsulation process. NTA was performed with the same procedure also on reduced anti-CD38 targeted liposome ZnO NCs (ZnO-LipCD38). TEM analyses were carried out on ZnO-Lip, according to the sample preparation process described above.

### Cell lines

B lymphocytes, IST-EBV-TW6B (IRCCS AOU San Martino), were cultured with Advanced RPMI 1640 (Gibco), supplemented with 20% heat-inactivated fetal bovine serum (FBS, Gibco) and 1% l-glutamine (Lonza). Burkitt’s lymphoma cell line, Daudi (ATCC, CCL-213), was cultivated with RPMI 1640 (ATCC) supplemented with 10% heat-inactivated fetal bovine serum (FBS, ATCC). Acute myeloid leukemia cell line, HL60 (ATCC, CCL-240), was cultivated in Iscove’s Modified Dulbecco’s Medium (Sigma) supplemented with 20% heat inactivated FBS (Sigma), 1% l-glutamine (Sigma). All the cell line media were completed with 100 μg/mL streptomycin and 100 units/mL penicillin (Sigma).

### Cytotoxicity and internalization tests

Cell viability was evaluated with WST-1 cell proliferation assay (Roche) after 24 h, and 48 h treatments with scalar doses of ZnO NCs, or liposome coated ZnO NCs (ZnO-Lip). ZnO NCs or ZnO-Lip nanoparticles were diluted in pre-warmed cell growth media to obtain different sample solutions with different concentration: 10, 20 and 40 μg/mL were tested. 2 × 10^4^ cells/well were seeded in 100 μL/well of prepared solution in 96 well flat bottom plates for suspension (Greiner-bio one) and incubated at 37 °C in 5% CO_2_. [39] After 20 h, or 44 h 10 μL of WST-1 reagent was added to each well, and after 4 h incubation WST-1 absorbance at 450 nm was detected by the Multiskan GO microplate spectrophotometer (Thermo Fisher Scientific) using 620 nm as reference. For the data analysis, the background signal of each treatment suspension is subtracted from the relative cell absorbance. The experiments were performed in duplicates.

The same procedure was employed to test cell viability of Daudi, Lymphocytes and HL60 after 24 h, or 48 h treatment with 40 μg/mL of liposome coated ZnO NCs (ZnO-Lip) and reduced anti-CD38 targeted liposome coated ZnO (ZnO-LipCD38). The experiments were performed in triplicates.

The internalization of ZnO-Lip and ZnO-LipCD38 was evaluated by flow cytometry, using a Guava Easycyte 6- 2 L instrument (Merck Millipore), as reported elsewhere [13, 28]. For this assay, after the lipidic-shell formation and NCs encapsulation, liposomes were labelled with DiD, using 5 μL of DiD for 1 mg of NCs and incubated with orbital shaking at 250 rpm at 37 °C for 30 min. 2 × 10^5^ cells/well were plated at 1 mL/well with cell medium containing 40 μg/mL of DiD labelled ZnO-Lip or DiD labelled ZnO-LipCD38 into 24 well flat bottom plates for suspension (Thermo Scientific) and incubated at 37 °C in 5% CO_2_. After 24 h, or 48 h cells were washed twice with PBS through centrifugation at 130*g* for 5 min, then re-suspended in 500 μL of PBS and analyzed. The experiments were performed in duplicates.

### Ultrasound treatments and evaluation of cell death

2 × 10^5^ cells/well were plated at 2 mL/well for US treatment in 24 well flat bottom plates for suspension (Thermo Scientific). The treated cells suspensions media were composed of: (i) cell growth media alone, to assess the cytotoxic effects of ultrasound (US), (ii) cell growth medium supplemented with 40 μg/mL of ZnO-Lip and (iii) cell growth medium supplemented with 40 μg/mL of ZnO-LipCD38. Plated cells were left at 37 °C in 5% CO_2_ for 24 h, and then exposed to ultrasound irradiation. US were generated by a LipoZero G39 (GLOBUS) at 1 MHz as frequency, 100% duty cycle, with different acoustic density generated by the transducer (0.3 W/cm^2^ and 0.45 W/cm^2^) and different exposure times (30 s and 1 min). Only two wells per plate were used, to avoid unwanted US exposure between the wells. Promptly after the US treatment, 100 μL/well of cells suspension was transferred into 96 well flat bottom plates for suspension (Greiner-bio one) and incubated at 37 °C in 5% CO_2_, with 3 technical replicates per sample. The combined cytotoxic effect of US and the nanoconstruct, and the cytotoxic effect of US alone were evaluated 24 h, and 48 h after the treatment, using the WST-1 cell proliferation assay, with the same procedure previously reported. The experiments were performed in triplicates.

Further analyses were performed to evaluate apoptotic processes which lead to the cytotoxicity of the combined US-nanoconstructs treatments. More in detail, cells were treated with US after their incubation with the nanoconstructs and incubated at 37 °C in 5% CO_2_. After 24 h, 100 μL of cell suspension was stained with 100 μL of Guava Nexin Reagent (Guava^®^ Nexin Reagent containing Annexin V and 7-AAD, Luminex), incubated at room temperature for 20 min. The resulting cell suspension was analyzed with the Guava Easycyte 6- 2 L instrument.

### Microscopy assays

Internalization of ZnO-Lip and ZnO-LipCD38 on Daudi and Lymphocyte cells was evaluated also through spinning-disk confocal fluorescence microscopy (Ti2 Nikon equipped with Crest Large FOV laser and 60 × PlanAPO objective, NA = 1.40). ZnO-Lip and ZnO-LipCD38 were labelled with DiD and 2 × 10^5^ cells/well were plated at 2 mL/well with different treatment suspension (controls, labelled ZnO-Lip, labelled ZnO-Lip CD38) in 24 well flat bottom plates for suspension, as reported for fluorimetric internalization assay. After 24 h of incubation at 37 °C in 5% CO_2_ cells were collected and centrifuged at 140*g* for 5 min and resuspended in 200 μL of PBS and then 200 μL of Image-iT Fixative Solution (4% formaldehyde, methanol-free, Thermo Scientific) was added, both PBS and fixative solution were at 4 °C. After 10 min of incubation, cells were centrifuged at 250*g* for 5 min and the pellet was resuspended in 400 μL of PBS. To label cell membrane, cells were centrifuged at 250*g* for 5 min and resuspended in 250 μL of PBS, and 0.6 μL of WGA conjugated with Alexa Fluor 488 (WGA488, λ_ex_ = 495 nm, Thermo Fisher) was added and let incubate for 10 min at room temperature. Cells were centrifuged again at 250*g* for 5 min, and resuspended in 250 μL of PBS, and 0.08 μL of Hoechst (Thermo Fisher Scientific) was added to label cell nuclei and incubate for 5 min at room temperature. Then, after two washing steps with PBS, cells are re-suspended in 250 μL of PBS and a 50 μL droplet of this cell solution was spotted in a 8-well chamber slide (Thermo Scientific Nunc Lab-Tek II CC2 Chamber Slide System) and confocal fluorescence microscopic images were acquired.

The effectiveness of the incorporation anti-CD38 fragments into the lipidic shell which cover ZnO NCs was evaluated through spinning-disk confocal fluorescence microscopy. First of all, ZnO-LipCD38 were incubated for 1 h at room temperature on a rotating wheel with 0.7 μL of secondary antibody (AffiniPure F(ab’)2 Fragment Goat Anti-Human IgG, Fc fragment specific, 1.3 mg/mL in water, Jackson Immunoresearch) bound to a coumarin fluorescent dye (λ_ex_ = 387). This secondary antibody strategy ensures the interaction just with Dartumumab fragments, due to the selectivity of the secondary antibody with the Fc portion of anti-CD38. Secondly, ZnO-LipCD38 were incubated with DiD, to label the liposome shell. Cells were than treated as reported above for the cytotoxicity test with 40 μg/mL of nanoconstruct. After 24 h cells were collected and resuspended in PBS and Fixative solution, as previously described, and cell membranes were labelled with WGA488 according to the procedure for the internalization studies.

To further investigate cell death mechanism after nanoconstruct and US exposure, fluorescence analysis were performed with wide-field inverted fluorescence microscope (Eclipse TiE from Nikon) equipped with 40 × objective (NA = 0.60). Cells were plated and treated as previously reported for the cytotoxicity assay. 24 h after the US exposure, cells were collected and free zinc (Zn^2+^) was labelled with FluoZin3-AM, resuspending cell pellets in 100 μL of 1 μM solution of FluoZin-3 AM fluorescent dye in cell culture medium and let incubate for 30 min at 37 °C. The cell membrane integrity was assessed with Propidium Iodide (PI, ThermoFisher) using 100 μL of 1 μM solution in cell culture medium for 5 min at 37 °C. Then, cells were washed two times with PBS, and finally resuspended in 200 μL of PBS. 50 μL of labelled cell suspension were spotted in a 8-well chamber slide (Thermo Scientific Nunc Lab-Tek II CC2 Chamber Slide System) and images were acquired and analyzed. The count of PI positive cells was performed separately from the count of FluoZin positive cells. For each experimental group of both cell lines, 5 images were acquired, and only viable cell were considered for counting.

### Statistical and synergy analysis

Data are expressed as mean ± standard error mean (SEM) and graphed with GraphPad Prism. Statistical analysis was performed by using One-Way Anova by GraphPad Prism *p < 0.0332, **p < 0.0021, ***p < 0.0002, ****p < 0.0001. The calculation of synergy scores and visualization of synergy maps was performed with SynergyFinder (https://synergyfinder.org), which is a free web-application for interactive analysis [40], using the Highest Single Agent (HSA) model. The heatmaps highlight synergistic and antagonistic dose regions in red and green colors, respectively. Synergy score can be: less than -10 (antagonistic interaction), between − 10 and 10 (addictive interaction), or larger than 10 (synergic interaction).

## Results and discussion

**Fig. 1:**
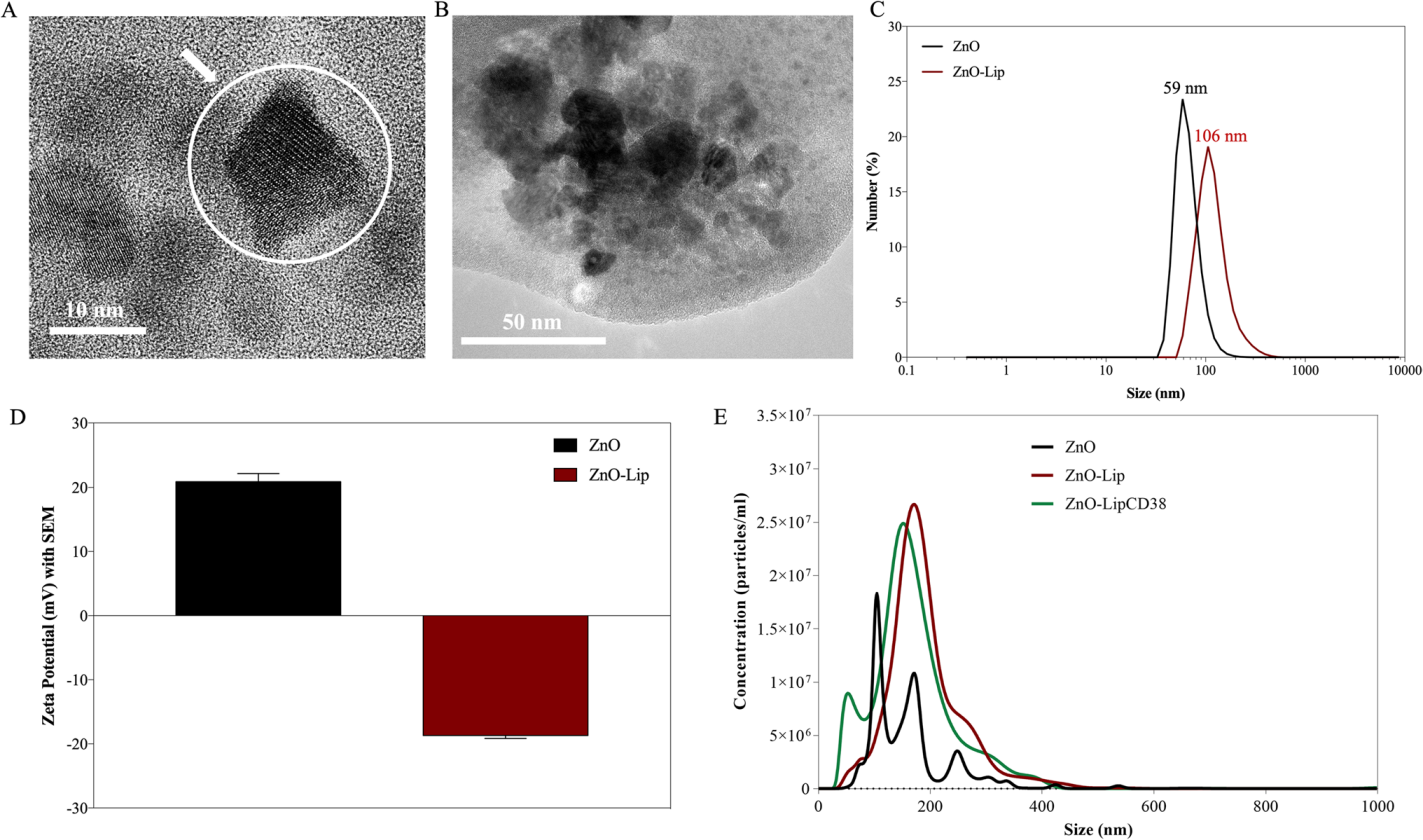
Transmission Electron Microscopic images of **a** ZnO NCs and **b** ZnO-Lip. **c** DLS measurements and **d** Z-potential of ZnO and ZnO-Lip **e** Nanoparticle Tracking Analysis measurements of ZnO (black curve), ZnO-Lip (red curve) and ZnO-Lip (green curve) in water

The synthesis of oleic-acid capped and amino-propyl functionalized ZnO NCs was performed by sol–gel synthetic route as reported in the Materials and Method section, leading to a colloidal suspension of nanoparticles. The morphology of ZnO NCs was assessed by High-Resolution TEM analysis. As shown in Fig. 1a, single nanocrystals with dimension ranging from 6 to 12 nm were successfully synthesized. The formulation of the lipidic mixture was ideated after considering the major features of natural extracellular vesicles (EVs), which possess an overall negative charge, a balanced composition of cholesterol and phospholipids showing the most abundant polar heads among phosphatidyl ethanolamine, phosphatidyl serine and phosphatidyl choline, and a different composition of saturated and unsaturated hydrophobic tales. Furthermore, EVs shows high stability and biocompatibility, as well as natural tropism towards specific recipient cells and ability to transport cargos and cross biological barriers, as recently reviewed [32]. The lipid bilayer composition here used is described in the Materials and Method section. The encapsulation of ZnO NCs into the lipidic shell is visible in Fig. 1b, revealing a detail of a liposomal-shell, which contains a cluster of several NCs. No kind of modifications in the morphology of ZnO were observed. It has to be noted that the analyzed sample of Fig. 1b was prepared letting it dry, without staining: this aspect can influence the shape of the lipidic vesicles on TEM analysis.

The lipidic shell formation can be supposed by DLS and Z-potential results, Fig. 1c, d respectively. The increase in hydrodynamic diameter in bd water from the ZnO (59 nm) to the ZnO-Lip formulation (106 nm) underlines the formation of the phospholipidic bilayer around the NCs, yet modifying the hydration layer of the particles and thus the hydrodynamic size in water. Due to the variation in the hydrodynamic diameter from ZnO to ZnO-Lip and the preparation process, the number of bilayers enveloping the ZnO nanocrystals can be supposed to be one or two.

Comparing the dimension of the single ZnO NC obtained by TEM analysis with both NTA (Fig. 1e, black line) and DLS (Fig. 1c, black line) results, a certain degree of aggregation can be hypothesized. The NTA shows in particular different size distribution peaks. However, the overall ZnO NCs colloidal stability is still good, considering the DLS data and the Z-Potential (Fig. 1d, black bar).

After the lipidic shell formation, a monodispersed size distribution is achieved, as measured by both DLS and NTA (red curves in Fig. 1c, e). Notably, the colloidal stability in water is greatly enhanced with respect to pristine ZnO NCs, as reported in Fig. 1e (red line), presenting a majorly monodisperse population despite the increase in the hydrodynamic diameter, the same result was previously obtained in RPMI [27]. Z-potential data, shown in Fig. 1d, further confirm the successful encapsulation of ZnO NCs in a lipidic shell, shifting from positive z-potential of ZnO NCs to negative values of ZnO-Lip. This change can be easily attributed to the main negative charge of the lipidic shell formulation. Additional experiments confirming the successful coating of ZnO NCs by the lipidic shell and the absence of liposomes without NCs are reported in Fig. SI-1 in the S.I., as Additional File.

In order to functionalize the liposome coating with targeting purposes, fragments of anti-CD38 were bounded to DSPE-PEG maleimide lipids, able to create, with the same solvent-exchange method, a targeted lipidic shell.

CD38 is a human type II transmembrane glycoprotein, widely expressed on multiple immune cell populations [41, 42]. The overexpression of CD38 on Daudi cells, in comparison to B Lymphocytes [43, 44], and the very low expression of CD38 on HL60, proposed here as a negative targeting control, are confirmed by CD38 expression measurements on Daudi, B-Lymphocytes and HL60, reported in the Supporting Information Fig. SI-2. This makes the CD38 a target of choice for developing the antibodies-targeted nanoconstruct. Daratumumab is an FDA approved anti-CD38 agent, which specifically and strongly binds to CD38 epitope on Daudi cell membrane. The Daratumumab reduction process allows to expose the thiol groups of anti-CD38, which can subsequently bind to DSPE-PEG maleimide. The success of Daratumumab reduction leading to different fragments can be deduced from the gel electrophoresis results reported in Fig. SI-3.

NTA analyses (Fig. 1e, green line) demonstrate that in presence of anti-CD38/DSPE-PEG maleimide, no major differences can be observed in the ZnO-LipCD38 with respect to the ZnO-Lip sample, suggesting the correct formation of the lipidic shell, encapsulating ZnO NCs and improving the colloidal stability of the nanoconstruct.

The lipidic shells enclosing ZnO NCs have multiple roles, beyond the increase of colloidal stability. Biocompatibility is enhanced, and the intrinsic biodegradable nature of lipids provide them with an augmented tolerance by human body [45]. Furthermore, liposomes are characterized by low toxicity, and this aspect can be exploited to deliver to cells a higher amount of ZnO NCs, which can be per se toxic in high doses, without causing any significant cytotoxic effects on in vitro cells due to the presence of the lipidic shell.

**Fig. 2:**
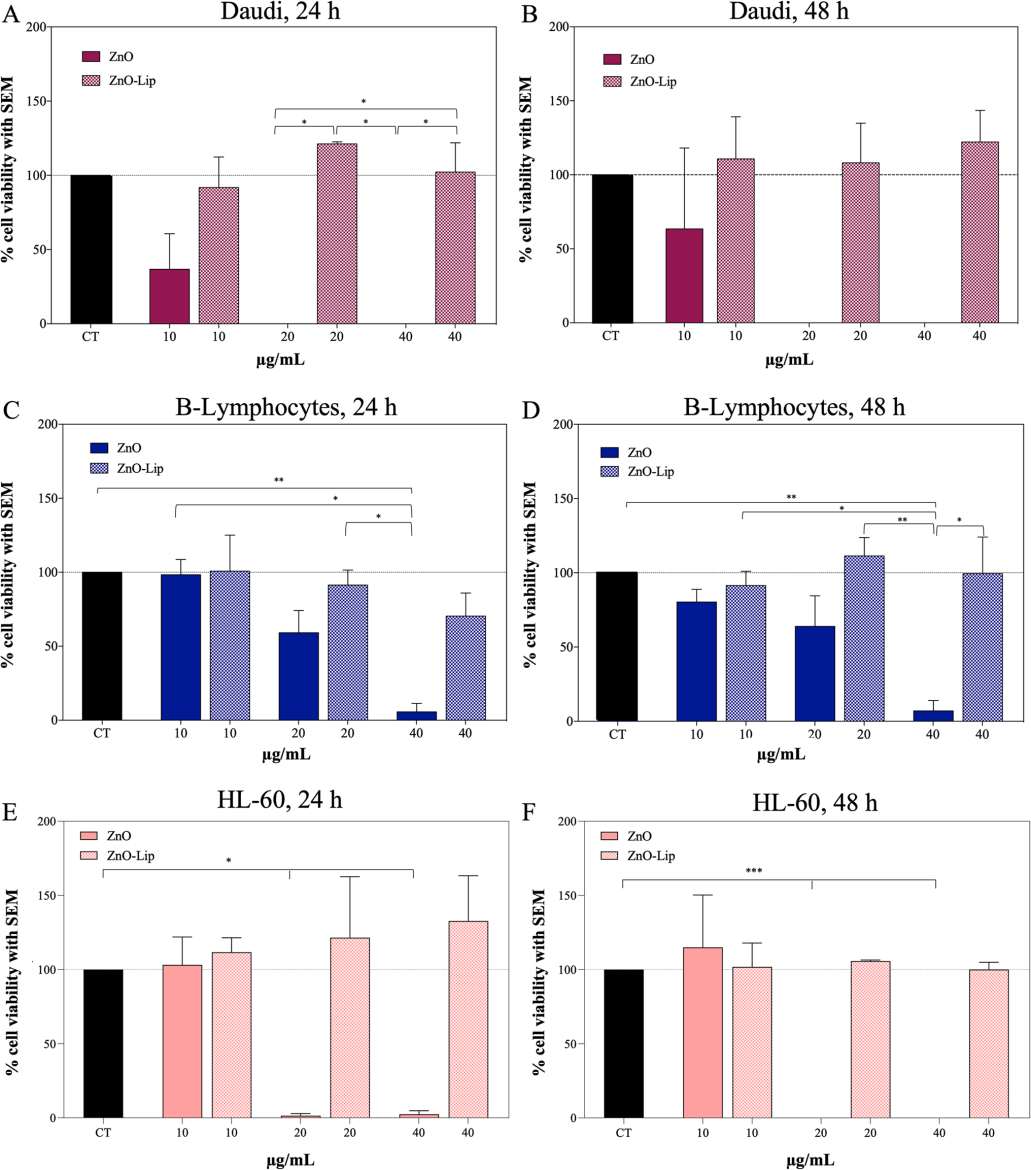
Cytotoxicity on Daudi cells at 24 h (**a**), and 48 h (**b**) of different concentrations of ZnO and ZnO-Lip. Cytotoxicity on Lymphocytes at 24 h (**c**), and 48 h (**d**) of different concentrations of ZnO and ZnO-Lip. Cytotoxicity on HL60 at 24 h (**e**), and 48 h (**f**) of different concentrations of ZnO and ZnO-Lip. All experiments were performed at least in duplicate. *p < 0.0332, **p < 0.0021, ***p < 0.0002, ****p < 0.0001

Figure 2 demonstrates the increased biocompatibility of ZnO-Lip with respect to pristine ZnO NCs. All the results are normalized with respect to the untreated control group (CT) for both Burkitt’s lymphoma cells, B-lymphocytes and myeloid leukemia cells. Different concentrations (i.e. 10, 20, 40 μg/mL) of ZnO and ZnO-Lip were administered to Burkitt’s lymphoma cells (Daudi, Fig. 2a, b), healthy counterparts (B-lymphocytes, Fig. 2c, d) and myeloid leukemia cells, characterized by a low CD38 expression (HL60, Fig. 2e, f) and the metabolic activity of cells was evaluated with WST-1 after 24 h (Fig. 2a, c, e), and 48 h (Fig. 2b, d, f). ZnO NCs result to be toxic for Daudi cells even at low dosage (10 μg/mL), and at higher concentration the complete death of Daudi population was detected. In contrast, significant differences are noticeable when ZnO-Lip were administered. For all dosages and at all time points, the cell viability of Daudi treated with ZnO-Lip is higher when compared to bare ZnO NCs, becoming significantly greater at higher dosages (both 20 and 40 μg/mL).

Different trends are demonstrated for B-Lymphocytes cells. The toxicity of ZnO NCs is lower per se, indeed a higher metabolic activity with respect to Daudi is measured for B-Lymphocytes at any time point, in particular for 10 and 20 μg/mL. Furthermore, ZnO-Lip results to be highly biocompatible at the tested concentration also in Lymphocytes. A significant difference is evident for 40 μg/mL, where Lymphocytes viability is lower than 10% when treated with ZnO and higher than 70% when ZnO-Lip where administered. Acute myeloid leukemia cells demonstrated an unvaried metabolic activity at 24 h (Fig. 2e) and an increased metabolic activity at 48 h (Fig. 2f) after the administration of 10 μg/mL of ZnO. Significant cytotoxic effects are visible at both 24 h (Fig. 2e) and 48 h (Fig. 2f) after the treatment with 20 μg/mL and 40 μg/mL of ZnO. Concerning the administration of ZnO-Lip, no significant variation in HL60 viability can be underlined for all the concentrations and both the time points.

These results suggest that even very low concentrations of ZnO NCs are characterized by an intrinsic killing selectivity toward Daudi and HL60 cells, with respect to the healthy counterparts, as reported in previous literature [16]. When high doses of ZnO NCs are administered, large cytotoxic effects occur in both cancer and healthy cells. This intrinsic cytotoxicity makes the role of lipidic shell crucial in administering higher doses of ZnO NCs while avoiding the cytotoxic effects. These results also highlight the high biocompatibility of the ZnO-Lip nanoconstruct, even at relatively high dosages.

**Fig. 3:**
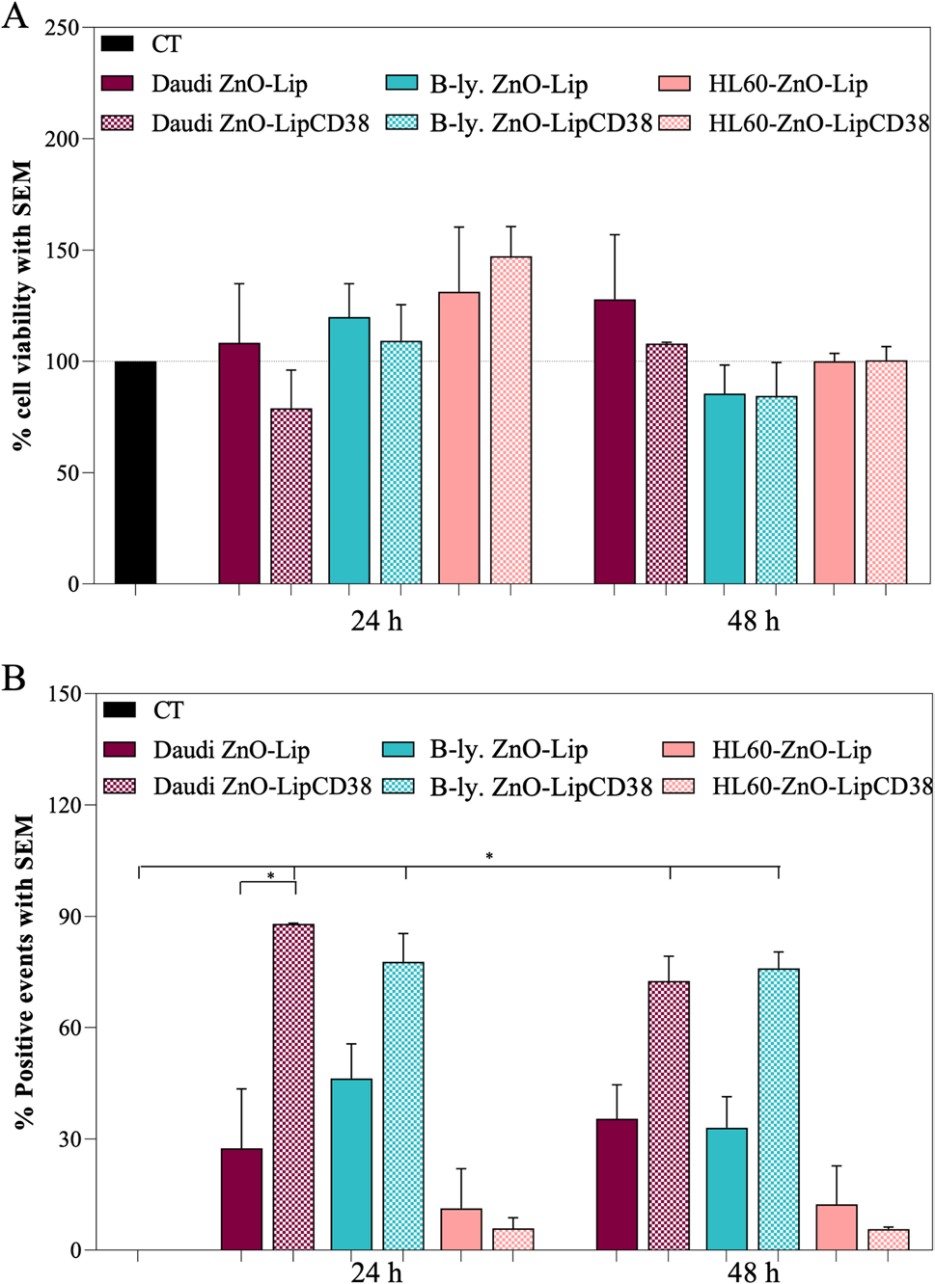
**a** Cytotoxcity on Daudi, B-Lymphocytes (B-ly.) and HL60 cell lines treated with 40 ug/mL of ZnO-Lip or ZnO-LipCD38 after 24 h and 48 h. **b** ZnO-Lip or ZnO-LipCD38 internalized or bounded to Daudi, Lymphocytes and HL60 cell membranes 24 h and 48 h after the treatment. Liposomes were marked with DiD, and cells were treated with 40 μg/mL of nanoconstructs. All experiments were performed at least in duplicate. *p < 0.0332, **p < 0.0021, ***p < 0.0002, ****p < 0.0001

Therefore, the dose chosen for further treatments was the highest one, i.e. 40 μg/mL of ZnO-Lip. The aim is thus to investigate the biological effects generated by the interaction of the nanosystem at the highest administered dose without significantly decrease of the viability in both Daudi and healthy B-Lymphocytes. The cell viability and internalization in both Daudi, HL60 and B-Lymphocytes were evaluated in presence or absence of anti-CD38 fragments conjugated to the lipidic shell of the nanoconstruct (i.e. ZnO-LipCD38 versus ZnO-Lip), as reported in Fig. 3a, b.

The cell viability of Lymphocytes is not affected by the administration of 40 μg/mL of neither ZnO-Lip and ZnO-LipCD38 at any studied time point. The same behavior can be noted for Daudi and HL60 cells, highlighting the excellent biocompatibility of the whole nanoconstruct, even in presence of Daratumumab fragments on the lipidic shell. Furthermore, the targeting action of anti-CD38 is demonstrated in Fig. 3b. The internalization results depicted in Fig. 3b consider as positive events both the nanoconstruct elements which are inside cells and the ones bounded to the external cellular membranes. The high internalization of ZnO-LipCD38 in both Daudi and B-Lymphocytes cell lines is visible at any time point, however the internalization of ZnO-LipCD38 is significantly higher with respect to the non-targeted counterpart in Daudi (*p* value equal to 0.0292) at 24 h. This result indicates that the CD38 antigen expression in both cell lines can be efficiently targeted by the fragmented antibodies and that these fragments are correctly bound and displayed at the lipidic shell surface. It is also important to observe that the significant increase of ZnO-LipCD38 nanoconstructs internalization in Daudi cell lines with respect to the non-targeted ZnO-Lip, is in contrast not significant for Lymphocytes. This result can be considered the effect of the higher overexpression of CD38 on the Daudi membrane with respect to the B-Lymphocytes. To further corroborate this evidence, the internalization of fragmented anti-CD38 targeted nanoconstructs is extremely and significantly lower for HL60 cells (Fig. 3b), which are CD38 negative. The decrease of internalization in CD38 negative cells underlines the efficacy of the targeting capability owned by the fragmented CD38 targeted NPs.

According to these results, an incubation time of the nanoconstruct equal to 24 h was chosen on both Daudi and Lymphocytes for further investigations and in particular to select the time point at which the external stimulus could be applied, to maximize the effect of ZnO-LipCD38 on Daudi cell line. The low internalization results achieved on HL60, provide the reason to not carry on any further experiments on this cell line.

**Fig. 4:**
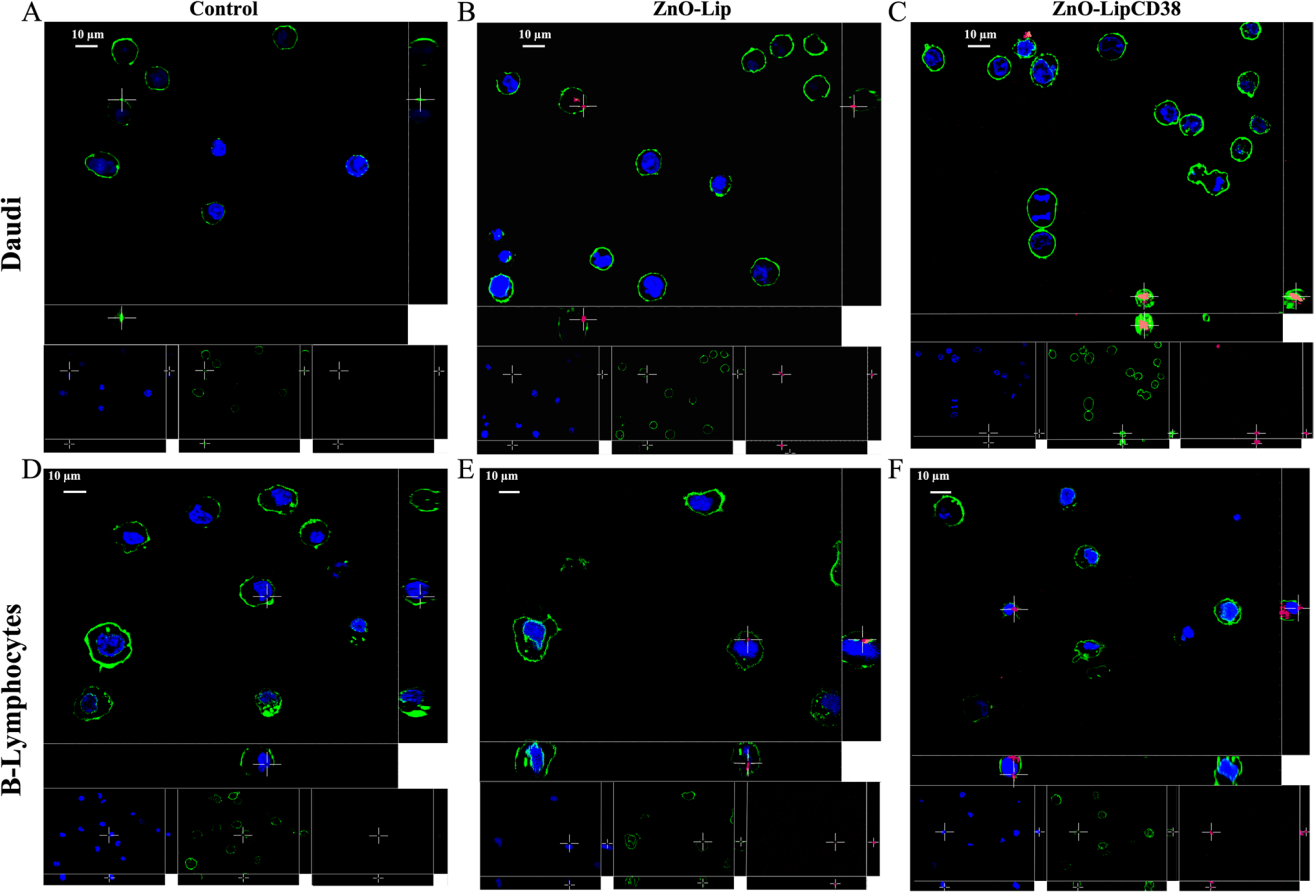
3D fluorescence microscopy images of Daudi (**a**) and lymphocytes (**d**) control cells, Daudi treated with ZnO-Lip (**b**) and ZnO-LipCD38 (**c**) and lymphocytes treated with ZnO-Lip (**e**) and ZnO-LipCD38 (**f**) after 24 h. Liposome containing ZnO NCs were labelled with DiD (red channel); cell nuclei were labelled with Hoechst (blue channel); cell membranes were labelled with WGA488 (green channel). Cells were treated with 40 μg/mL

Further analyses were performed after 24 h from the administration of nanoconstructs (40 μg/mL). 3D fluorescence images of Daudi and B-Lymphocytes were acquired after the incubation with ZnO-Lip and ZnO-LipCD38. Cell membranes were provided with fluorescence via WGA 488 (green elements), cell nuclei were labelled with Hoechst (blue elements) and the liposomes, both targeted and not targeted, were labelled with DiD (red elements). The internalization is verified in both cell lines, and the 3D fluorescent images reported in Fig. 4 show that the nanoconstructs are found to be bounded to the external cellular membrane or inside the cellular membrane, but outside the cell nuclei. It is possible to distinguish an increased amount of internalized red elements, which represent the nanoconstructs, when targeted ZnO-LipCD38 was administered to cells, in agreement with the internalization results obtained with flow cytometry in Fig. 3b.

Fluorescent microscopy was also exploited to ensure the presence of the fragmented antiCD38 in the liposome containing ZnO NCs after the cell internalization.

**Fig. 5:**
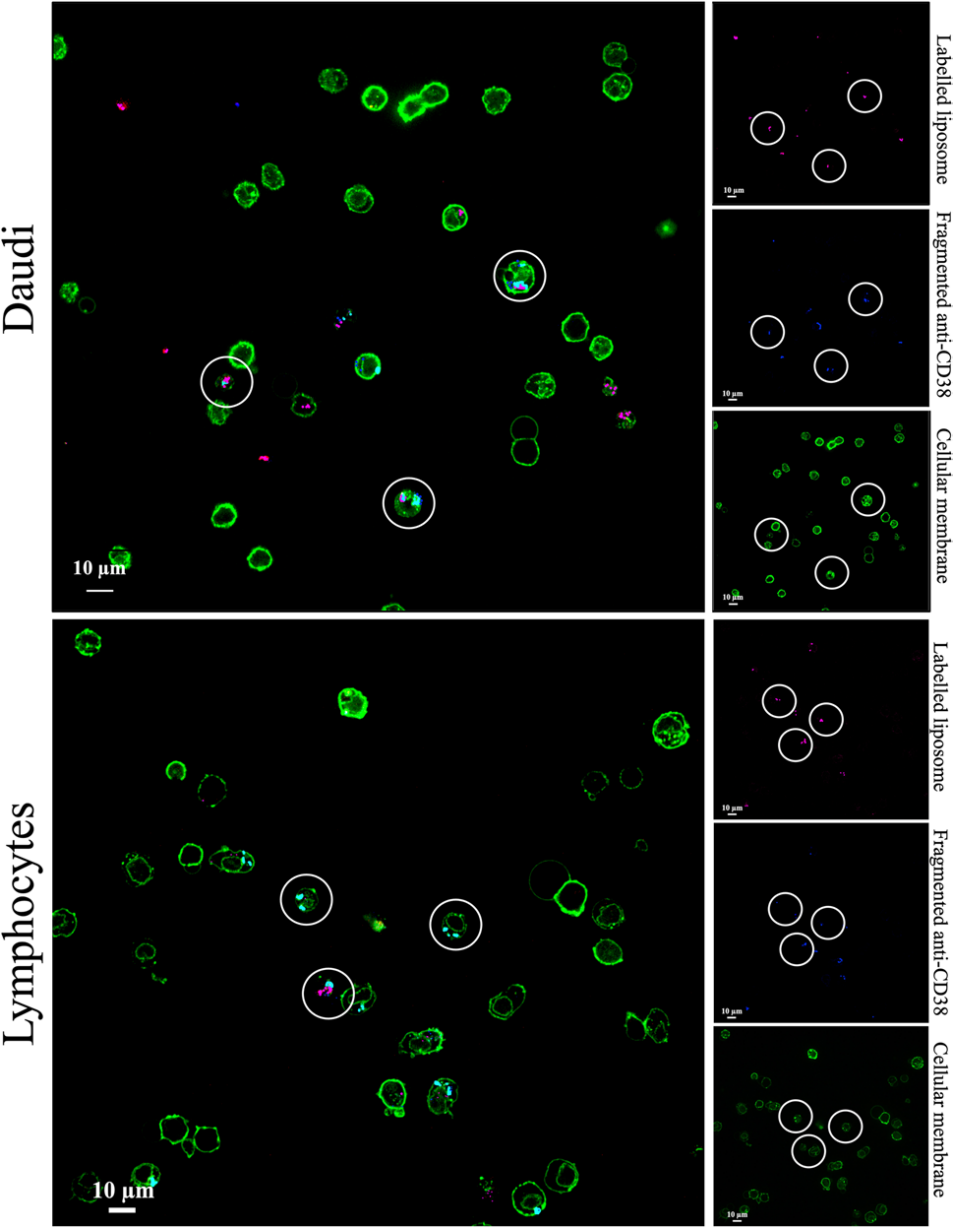
Fluorescence microscopy images of the internalization and colocalization of ZnO-LipCD38 nanoconstruct on Daudi and lymphocytes after 24 h. Liposome containing ZnO NCs were labelled with DiD (red channel); antiCD38 fragments incorporated in the lipidic shell contained ZnO NCs were labelled with Curcumin (blue channel); cell membranes were labelled with WGA488 (green channel). Cells were treated with 40 μg/mL. White circles represent highly relevant internalization events in cells

In particular, Fig. 5 demonstrates the colocalization of anti-CD38 fragments and the rest of the lipidic cells, and at the same time it shows the internalization in cells. The liposomes were labelled with DiD (pink dots, first column), while anti-CD38 fragments where selectively labelled by a secondary antibody conjugated with Coumarin dye (blue dots) and cell membrane was stained with WGA 488 (green elements). The larger images of Fig. 5 represent the merging of all the three fluorescent channels, and they establish, for both Daudi and Lymphocytes, that the nanoconstruct is inside the cell membrane. The same images also verify the correct incorporation of antiCD38/DSPE-PEG lipid in the lipidic shell during the liposome formation process around ZnO NCs.

After the ZnO-Lip and ZnO-LipCD38 characterization, the assessment of non-toxic dose (40 μg/mL) on both Daudi and B-Lymphocytes, and the correct incubation time in cells to maximize further effects, then the combination of ZnO-LipCD38 and US exposure was investigated.

As previously reported in the literature[12, 46–49], ultrasounds produce different thermal and non-thermal effects, making the sonodynamic therapy an emerging approach for non-invasive treatment of less accessible lesions or tumors [50]. More in details, the periodical pressure waves provided by ultrasound irradiation can induce inertial cavitation, producing ROS and a large amount of mechanical stress and stream jests, which can all contribute to damage to the nearby elements, such as cells. The presence of nanoparticles introduces a higher number of nano bubbles, trapped on their surface, and, combined to US, can decrease the threshold for which inertial cavitation can occur [51, 52], permitting the decrease of the administered US dose and therefore the reduction of unwanted side effects, as the thermal damages in healthy tissue. ZnO represents a suitable candidate for its ability to respond to US, for its bioimaging potentialities and for its piezoelectric properties [12] and for its chemical instability at low pH. In addition, considering in vitro systems, the physical movement of nanoparticles internalized into cells under US exposure could also contribute to the cell death, locally increasing the temperature and leading to the mechanical destruction of cells [13].

Therefore, ultrasounds were combined with ZnO-Lip and ZnO-LipCD38 dose (40 μg/mL) and the effects on both Daudi and B-Lymphocytes cell lines were evaluated 24 h, and 48 h after the treatment with US. Different US input power densities (0.3 and 0.45 W/cm^2^) and different exposure times (30 s and 1 min) were tested under continuous mode and with a planar US transducer (see more details in the Materials and Method section).

**Fig. 6:**
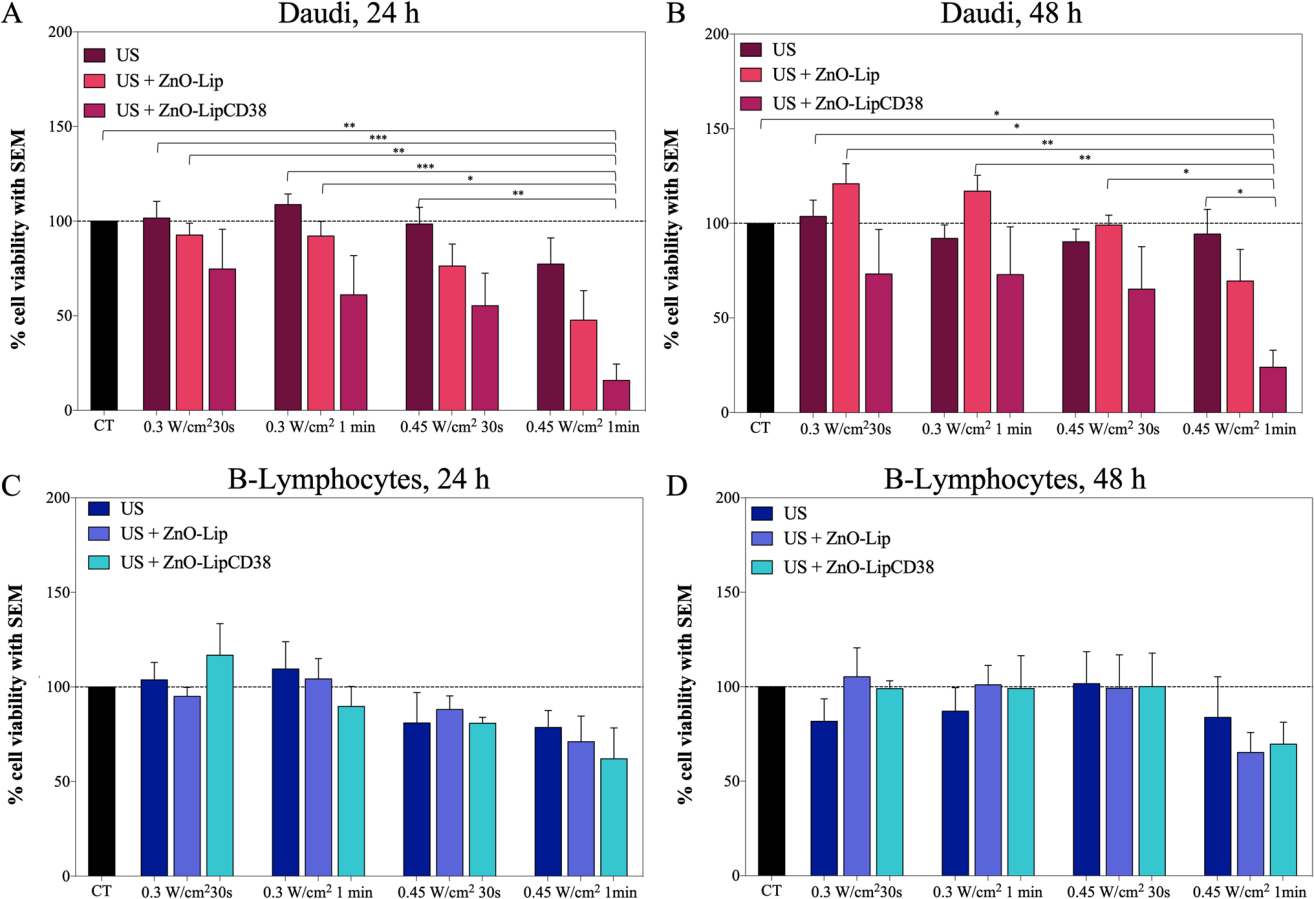
Cells viability of Daudi after 24 h (**a**) and 48 h (**b**) the treatment with US; Lymphocytes viability after 24 h (**d**) and 48 h (**e**) the treatment with US. US were produced by Lipo0 transducer, at 1 MHz, 100%DC. All the experiments were performed at least in triplicates. In US + ZnO-Lip and US + ZnO-LipCD38 groups, cells were previously incubated with 40 μg/mL of nanoconstructs 24 h before the US treatment. *p < 0.0332, **p < 0.0021, ***p < 0.0002, ****p < 0.0001

As visible in Fig. 6a, US alone result to be non-toxic for Daudi cells after 24 h and 48 h from the treatments. The same result is obtained for B-Lymphocytes at all time points, suggesting that all tested doses of US do not cause any cytotoxic effect on cells. This allows to consider them as safe per se. After 24 h, the combination of ZnO-Lip and US can still be considered safe in Daudi for both 30 s and 1 min time exposures at 0.3 W/cm^2^, and for 30 s exposure at 0.45 W/cm^2^ (Fig. 6a). In contrast, the cell metabolic activity starts to decrease for 1 min exposure at 0.45 W/cm^2^ in combination with ZnO-Lip. A recovery trend is visible after 48 h (Fig. 6b) for Daudi cells which received ZnO-Lip and 0.45 W/cm^2^ for 1 min.

Noteworthy, the cytotoxic effect of ZnO-LipCD38 and US is more remarkable, starting from 1 min exposure at 0.3 W/cm^2^ at 24 h (Fig. 6a). At 48 h after the treatment with 0.45 W/cm^2^ and ZnO-LipCD38 (Fig. 6b), a significant difference is obtained between Daudi treated with US and the ones treated with the combination of US and targeted nanoconstruct. No significant difference is found with US and non-targeted nanoconstructs at the same conditions.

The B-Lymphocytes viability was evaluated at 24 h (Fig. 6c), and 48 h (Fig. 6d) after the different US treatments in combination with ZnO-Lip and ZnO-LipCD38. A small cytotoxic effect can be seen at 24 h for B-Lymphocytes treated with 0.45 W/cm^2^ for 1 min in combination with both ZnO-Lip and ZnO-LipCD38. However, no significant difference was obtained in B-Lymphocytes viability for all the different treatment combinations, nor for the different time points.

These results underline the effective killing capability of the combination of 1 min US exposure at 0.45 W/cm^2^ and ZnO-LipCD38 solely towards Daudi cells, leaving the healthy counterparts, i.e. B-Lymphocytes, without any significant cytotoxic effect.

**Fig. 7:**
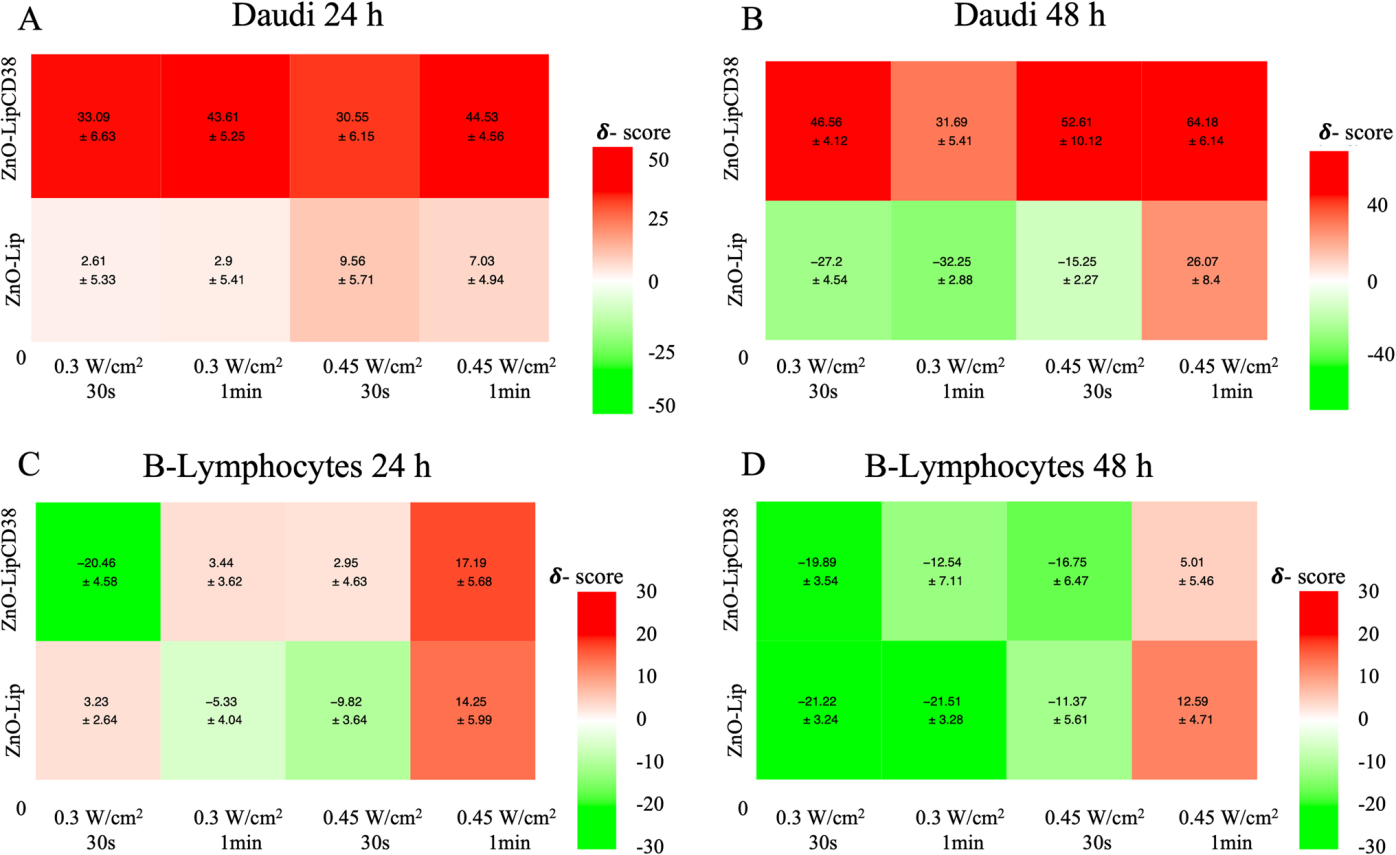
Evaluation of the synergy derived from the combination of ultrasound irradiation and the administration of ZnO-Lip and ZnO-LipCD38 on Daudi, at 24 h (**a**) and 48 h (**b**), and on B-Lymphocytes, at 24 h (**c**) and 48 h (**d**). The effects generated by the administration of the two treatments (US and ZnO-Lip/ZnO-LipCD38) can be evaluated by the synergy score (δ); it can be < − 10 and it represents antagonistic interaction, > − 10 and < 10 and it represents additive interaction, or it can be > 10 and it represents synergistic interaction. The heatmaps highlight synergistic and antagonistic dose regions in red and green colors, respectively. Cells were previously incubated with 40 μg/mL of nanoconstructs 24 h before the US treatment The analysed data were at least in triplicate

To deeply study the type of in vitro interaction occurred between ultrasound stimulation and both ZnO-Lip or ZnO-LipCD38, the viability data reported in Fig. 6 were analyzed with synergyFinder software [40]. The results of these analysis are reported in Fig. 7.

The combination of ultrasound, especially at 0.45 W/cm^2^ with 1 min exposition, and ZnO-LipCD38 result to be synergic for determining the Daudi cell death at both 24 h and 48 h, with a synergy score (δ) larger than 40, as visible in Fig. 7a, b. For these cancer cells, every combination of US and nanoconstruct can be considered as a synergic treatment (red areas). A different scenario is depicted for healthy B-lymphocytes in Fig. 7c, d.

Here, the same conditions of US are characterized by very small synergic scores, representing the antagonistic action of US and nanoconstructs. Only B-lymphocytes treated with 0.45 W/cm^2^ for 1 min and ZnO-LipCD38 were affected by synergic effects, even if the synergic score for this condition is less than half of the one obtained for Daudi cell. Especially after 48 h from the US treatment (Fig. 7b), the combination of US and nanoconstructs can be considered antagonist for almost any condition (green area). These results underline once more the effectiveness of the synergic treatment here proposed and its selectivity, having no killing effects on the healthy counterpart, i.e. B-Lymphocytes.

To further investigate the killing mechanism, a dual fluorescent probe strategy test was performed on Daudi and B-Lymphocytes treated with 1 min exposure at 0.45 W/cm^2^ and ZnO-Lip or ZnO-LipCD38. Annexin V-PE is a fluorescent dye able to monitor the externalization of phosphatidylserine, which is translocated to the outer of cell membrane during early-stage apoptosis [53]. On the other hand, 7-AAD is a fluorescent DNA intercalator, which can permeate the cell membrane of late-stage apoptotic or death cells [54].

**Fig. 8:**
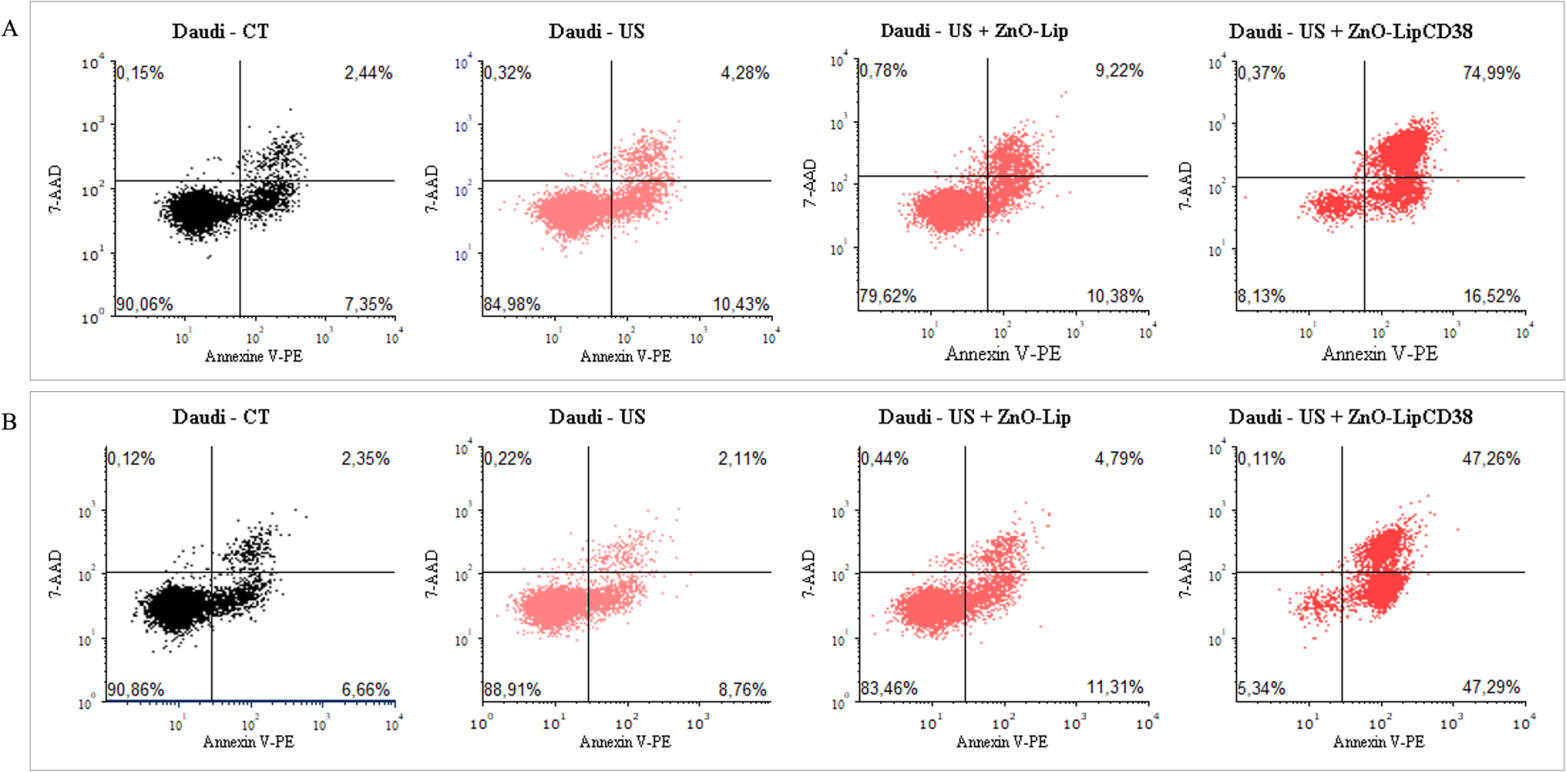
Evaluation of percentage of early and late apoptotic populations after 24 h (**a**), and 48 h (**b**) induced in Daudi cells after the US treatment with Lipo0 transducer (1 MHz, 100%DC, 0.45 W/cm^2^, 1 min). In US + ZnO-Lip and US + ZnO-LipCD38 groups, cells were previously incubated with 40 μg/mL of nanoconstructs 24 h before the US treatment

**Fig. 9:**
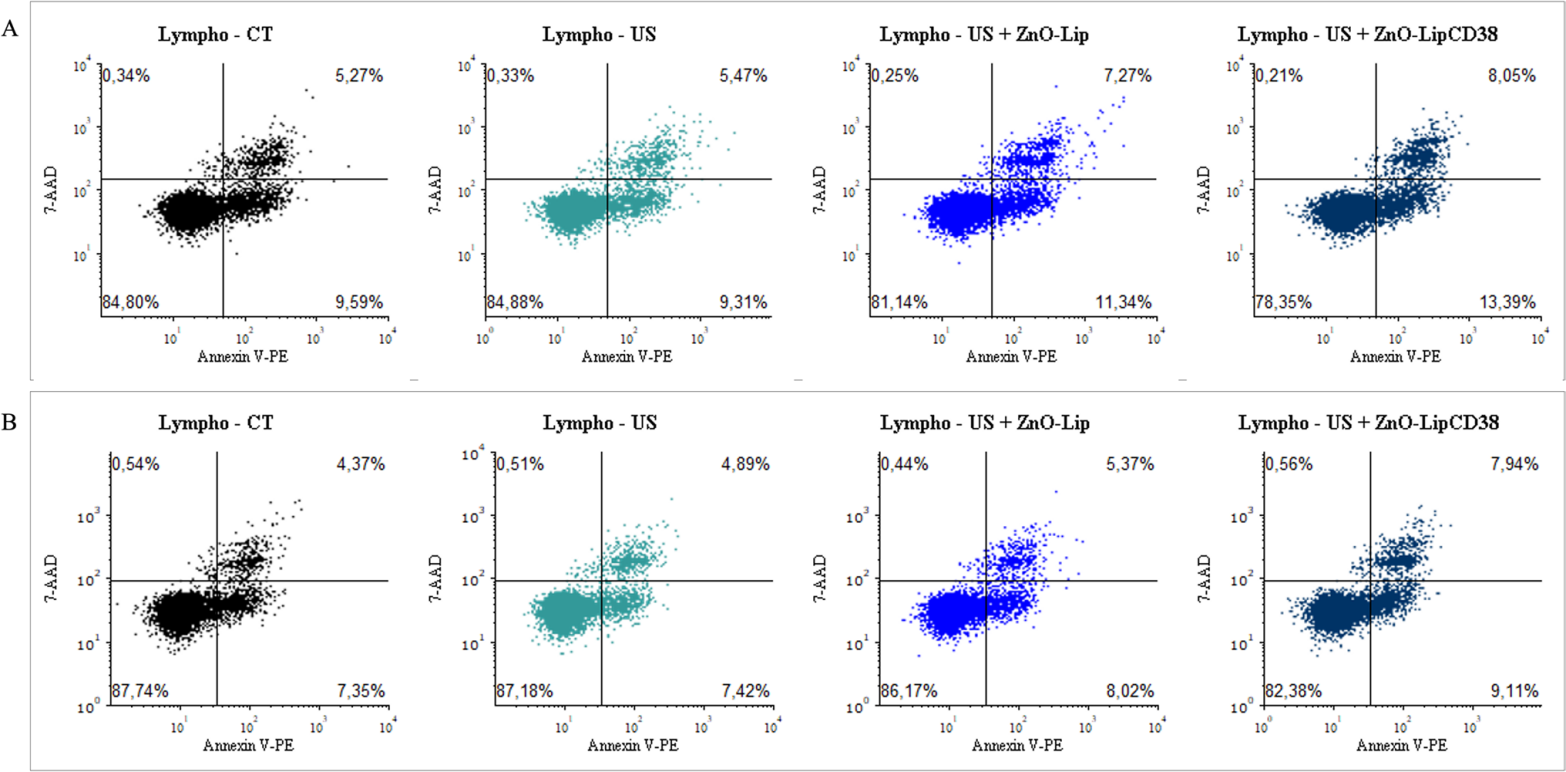
Evaluation of percentage of early and late apoptotic populations after 24 h (**a**), and 48 h (**b**) induced in B-Lymphocytes cells after the US treatment with Lipo0 transducer (1 MHz, 100%DC, 0.45 W/cm^2^, 1 min). In US + ZnO-Lip and US + ZnO-LipCD38 groups, cells were previously incubated with 40 μg/mL of nanoconstructs 24 h before the US treatment

Outcomes of this assay are displayed in a dot-plot with quadrant-defined population. Indeed, it is possible to distinguish 4 different cell populations: non-apoptotic cells (lower left quadrant), early apoptotic cells (lower right quadrant), late stage apoptotic and dead cells (upper right quadrant), and cell debris (upper left quadrant). Results are shown in Fig. 8 for Daudi and in Fig. 9 for B-Lymphocytes, data were collected at 24 h (Figs. 8a, 9a), and 48 h (Figs. 8b, 9b) after the US treatments.

At 24 h, Daudi cells (Fig. 8a) were not affected by US alone and by US in combination with ZnO-Lip, but when cells were treated with US and ZnO-LipCD38, an increase in both early-stage and late-stage apoptosis is visible. More in details, for the US + ZnO-LipCD38 group, 16.5% and 75% of cell population is at early-stage and late-stage apoptosis, respectively. In contrast, for both US and US + ZnO-Lip groups just ~ 10% of cell population is in early-stage apoptosis and lower % are recorded for late apoptosis. These results suggest that, during the first 24 h, at least 75% of cell population underwent to an apoptosis process. This process can be promoted by the mechanical stress and nano-scalpel effects [13] caused by inertial cavitation during the US exposure. Instead, after 48 h (Fig. 8b) there is a progressive increase of early-stage apoptosis cells for US + ZnO-LipCD38 group, which becomes 47% of cell population at 48 h. In contrast, the early-stage apoptosis cell population remains stable for all the other groups at any time points. It can be assumed that the Daudi population which survived to nanoconstruct + US induced damages after 24 h from the treatment with US + ZnO-LipCD38, activate pathways of programmed cell death, rising the killing efficacy of the synergy composed by external stimulus and the targeted nanoconstruct.

The results presented in Fig. 9 for B-Lymphocytes are in accordance with the previously presented data. The percentage of both early-stage and late-stage apoptotic cells for US, US + ZnO-Lip and US + ZnO-LipCD38 groups are similar to the physiological values visible in the untreated cells (black dot plot, control cells) at any time point (Fig. 9a, b). These data suggest one more time the safety of the US-activated and targeted nanconstruct treatment on healthy cells, while underline its effectiveness of Burkitt’s lymphoma cells. CD38-targeted liposome containing ZnO NCs is thus proved to be externally activated by US stimuli and to selectively damage only cancerous cells.

**Fig. 10:**
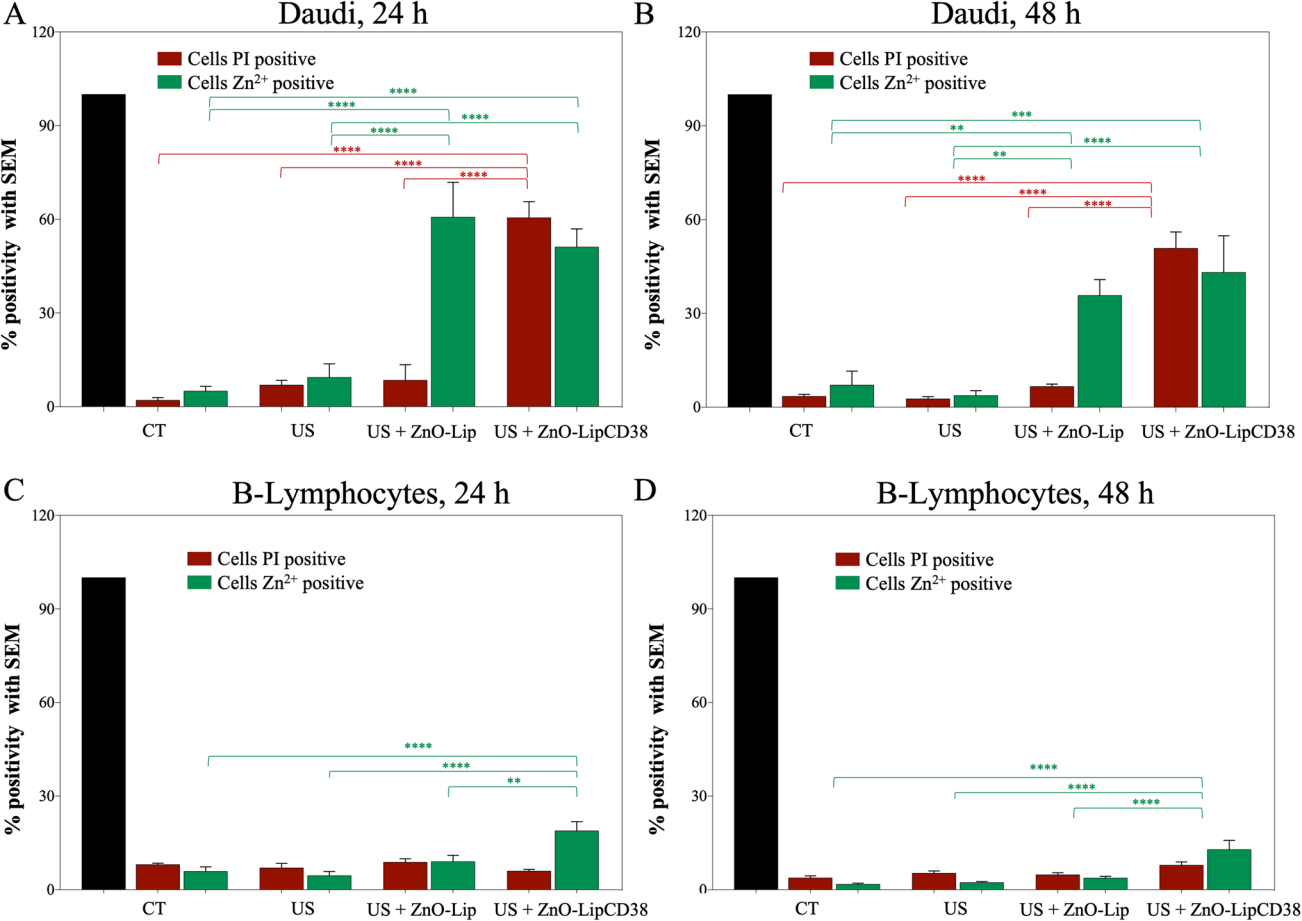
Cells membrane integrity was evaluated with PI dye, while Zn^2+^ presence inside cell was measured by Fluozin dye. Percentage of cells positive to PI and Fluozin is shown for Daudi cells at 24 h (**a**), and 48 h (**b**) after treatment with US, and for B-Lymphocytes after 24 h (**c**), and 48 h (**d**) the US treatment. US were produced by Lipo0 transducer, at 1 MHz, 100%DC, 0.45W/cm^2^, 1 min. 5 images per sample were considered, the two staining were evaluated separately. All the data are normalized with respect the total number of cells evaluated for each image (black column). In US + ZnO-Lip and US + ZnO-LipCD38 groups, cells were incubated with 40 μg/mL of nanoconstructs 24 h before the US treatment. *p < 0.0332, **p < 0.0021, ***p < 0.0002, ****p < 0.0001

A further demonstration of the achieved results is presented in Fig. 10a, b, for Daudi cells at 24 h, and 48 h, respectively and in Fig. 10c, d for B-Lymphocytes at 24 h, and 48 h. Cells were stained with two different fluorescent dyes: FluoZin-3AM dye which labels intracellular Zn^2+^ ions [55, 56], and Propidium Iodide (PI) which is a DNA intercalator, impermeable to intact cell membrane [57, 58] used here as detector for damaged cell membranes. Representative images are further reported in Figure SI-4.

The presence of Zn^2+^ ions is significantly higher in Daudi and in Lymphocytes which received either ZnO-Lip or ZnO-LipCD38 at any time point, as expected. However, the percentage of positive events is above 50% for Daudi, while is below 30% for B-Lymphocytes pointing out that the ZnO-Lip construct is totally safe for B-Lymphocytes, sparing them also from zinc cation cytotoxic effects. The damages in the cell membrane reflect the results obtained with all the other techniques. A significantly higher amount of PI signal was detected in Daudi cells treated with US + ZnO-LipCD38 with respect to both US and US + ZnO-Lip groups 24 h and 48 h after the treatment (Fig. 10a, b, respectively). It can be assumed that the higher cell membrane damage is directly related to the efficacy of the combined targeted therapy [59, 60]. Instead, no significant results are obtained for B-Lymphocytes, in which the percentage of cells positive to PI remains close to the one measured for control (untreated) cells.

It has been reported in the previous literature [11, 12, 18], the enhanced ROS production generated by the ultrasound irradiation of ZnO nanoparticles, due to the increased number of inertial cavitation nuclei at NPs surface. ROS are involved in various cell signaling processes [61], and an imbalance in cellular redox homeostasis could cause cellular component damages, as well as the activation of many signal pathways liable of cell death, causing ROS-mediated apoptosis or necrosis [62]. Oxidative stress is not the only cytotoxic effect which compete to cells death, but also stress, mechanical damages, and toxic Zn^2+^ release, as confirmed by Fig. 10, could be responsible for the decrease of cellular viability after the exposure to the targeted nanoconstruct and US irradiation. The results presented in Figs. 9 and 10c, d suggest that the cellular metabolism of B-Lymphocytes is less sensitive to the mingling of consequences derived from US irradiation and ZnO-LipCD38 administration with respect to cancerous Daudi cells. The overall outcome is the targeted cytotoxicity of the proposed strategy toward Burkitt’s lymphoma cells, without any significant effect on the healthy counterpart.

## Conclusion

In this paper a liposome-based shielding approach for ZnO NCs, and a successful decoration with fragmented anti-CD38 for targeting purposes were established. The effective lipidic shell formation around NCs has here proved to be effective in enhancing the colloidal stability of the nanoconstruct and diminishing the cytotoxicity of pristine ZnO NCs in hematological cells, using two types of cancer cells lines and healthy ones. Fragmentation of clinical-grade Daratumumab occurred in a controlled manner, allowing to bound fragments of reduced anti-CD38 with DSPE-PEG Maleimide lipids, permitting their incorporation during the self-assembly process of liposome formation around ZnO NCs. The augmented internalization of the targeted nanoconstruct in Burkitt’s lymphoma cells in comparison to the one in healthy B-Lymphocytes and in a CD38^−^ acute myeloid leukemia (HL60) demonstrates the targeting efficiency of anti-CD38 lipid-coated ZnO NCs.

Further steps were taken towards the therapeutic applicability of the nanoconstruct for antitumoral purposes to obtain an efficient Trojan nanohorse. CD38-targeted nanocostructs were proven as potential therapeutic agent in synergy with ultrasound exposure with Burkitt’s Lymphoma cell line, demonstrating their striking effectiveness. Furthermore, the nanoconstruct was proven to be highly biocompatible when inactivated, and to possess high killing capability when US-activated only towards cancer cells, leaving healthy B-Lymphocytes cells without any significant damage. These results open the possibility of future applications of the nanoconstruct in vivo*,* particularly for the high specificity in targeting only towards selective cancerous cells, avoiding unwanted damage to healthy cells.

### Supplementary Information

**Additional file 1**.

## Data Availability

The datasets used and/or analysed during the current study are available from the corresponding author on reasonable request.
