# Supplementary material for: Anti-CD38 targeted nanotrojan horses stimulated by acoustic waves as therapeutic nanotools selectively against Burkitt’s lymphoma cells
Source: Discov Nano. 2024 Feb 14;19(1):28. doi: 10.1186/s11671-024-03976-z (PMC10866835; doi:10.1186/s11671-024-03976-z)
Supplement: Supplementary file 1 — Additional file 1. [file 11671_2024_3976_MOESM1_ESM.docx]

Anti-CD38 Targeted NanoTrojan Horses Stimulated by Acoustic Waves as Therapeutic Nanotools selectively against Burkitt’s Lymphoma Cells

Veronica Vighetto^1^, Marzia Conte^1^, Giada Rosso^1^, Marco Carofiglio^1^, Federica Sidoti Abate^1^, Luisa Racca^1^, Giulia Mesiano^1^, Valentina Cauda^1*^

^1^ Department of Applied Science and Technology, Politecnico di Torino, 10129, Torino, Italy

^*^ Corresponding author:

Tel. : +39 011.0907389

e-mail: valentina.cauda@polito.it

**Supporting Information**

**1. Colocalization study via fluorescence microscopy**

Figure SI-1 shows the signal of the far-red channel (left image) due to the emission of the ZnO NCs covalently labeled with Atto647 dye. The signal from the green channel (image in the center) shows the green emitting signal of the DiO lipophilic dye conjugated to the lipidic shells. The two channels are almost fully colocalized, with a calculated colocalization percentage of 94%, as visible in the merged channel (right image).


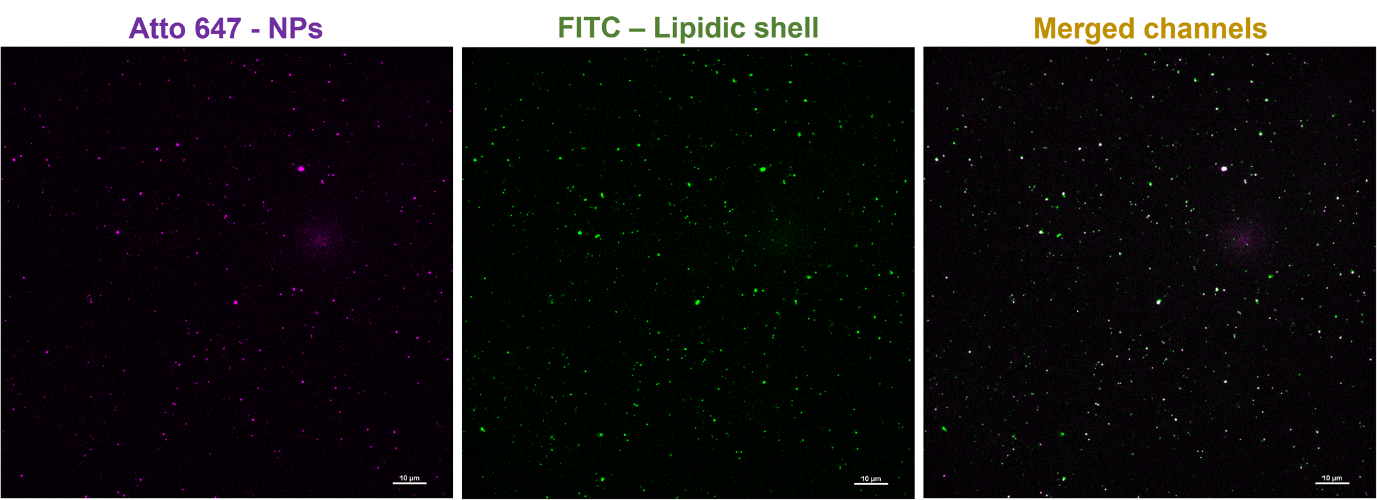


**Figure SI-1.** Fluorescence microscopy images showing the colocalization of the signal corresponding

to the NPs (Atto647, far red channel, image on the left) with the signal corresponding to the lipidic shell (DiO, green channel, image in the center) in the merged channels on the right. Scale bars are of 10 µm.

**2. CD38 expression on cells**

The expression of CD38 antigen on cell membrane surfaces was evaluated for all the cell lines.

Cells were collected and centrifuged at 150 g for 5 min and were resuspended in 1 mL PBS/0.5% BSA, as recommended by the manufacturer. Cells were incubated for 10 min in the dark at 4° degree with CD38 antibody REAfinity (Miltenyi Biotec) and the respective isotype control. Cells were washed twice, and then resuspended in 1 mL PBS/0.5% BSA. Unstained cells were used as a control population to exclude debris and impurity derived from buffer solution.


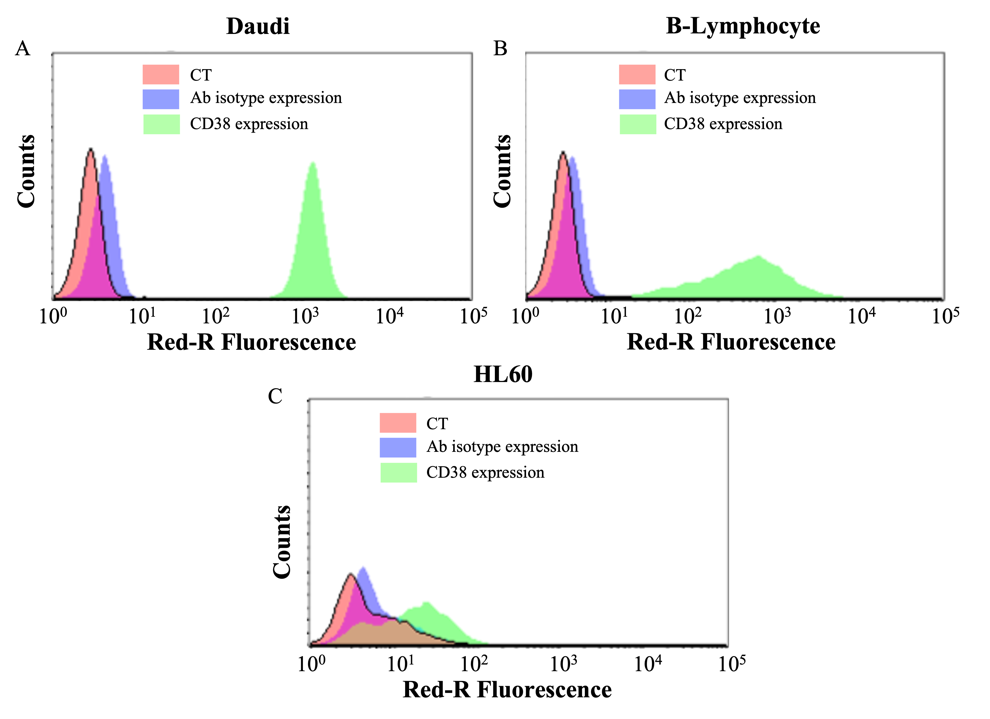


**Figure SI-2**. CD38 expression on Daudi (A), B-Lymphocytes (B) and HL60 (C) cells’ membrane. Evaluation performed with flowcytometry technique.

The overexpression of CD38 on Daudi cells in comparison to B-Lymphocytes is confirmed by CD38 expression measurements.

HL60 is CD38 negative cell line, and as visible in Figure SI-1 the signal derived from CD38 expression is low, almost superimposed to the isotype antibody signal.

**2. AntiCD38 reduction**

The success of Daratumumab reduction leading to different fragments can be deduced from the gel electrophoresis results reported in SI-3.


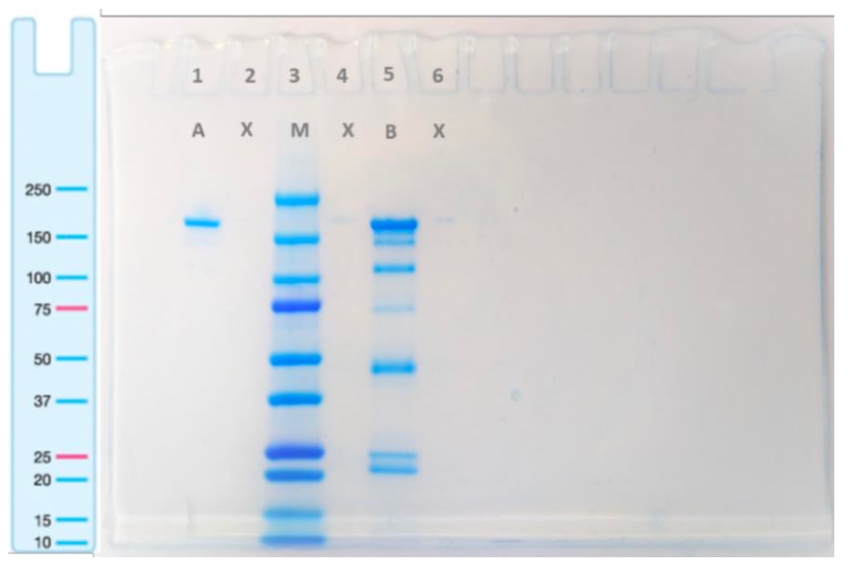


**Figure SI-3**. SDS-PAGE of anti-CD38 reduction. Non-reduced Daratumumab is represented in column A, reduced anti-CD38 in column B, while the marker corresponds to column M. scale unit: kDa.

The Daratumumab reduction process allows to expose the thiol groups of anti-CD38, which can subsequently bind to DSPE-PEG maleimide.

**4. PI – FluoZin3-AM staining on treated cells**

Daudi and B-lymphocytes were stained with PI and FluoZin3-AM to evaluate membrane integrity and Zn^2+^ presence, respectively. The staining was performed 24h and 48h after the treatment with US. The number of cells positive to PI was evaluated separately from the number of cells positive to Fluozin. Figure SI-4 shows the images of cells after 24 h from the treatment with US. Daudi cells treated with US + ZnO-Lip and US + ZnO-LipCD38 are visibly less in number, and for Daudi cells treated with US + ZnO-LipCD38 it is evident that the viability of the remaining cells is low (due to debris presence and absence of clear round shape of cells). B-lymphocytes showed in Figure SI-4 preserve their shape and integrity throughout all the experimental groups, even though there are less cells in the sample treated with US + ZnO-LipCD38. The results obtained from this analysis are in line with the viability results of Daudi and B-lymphocutes shown in Figure 6 of the main text.

**
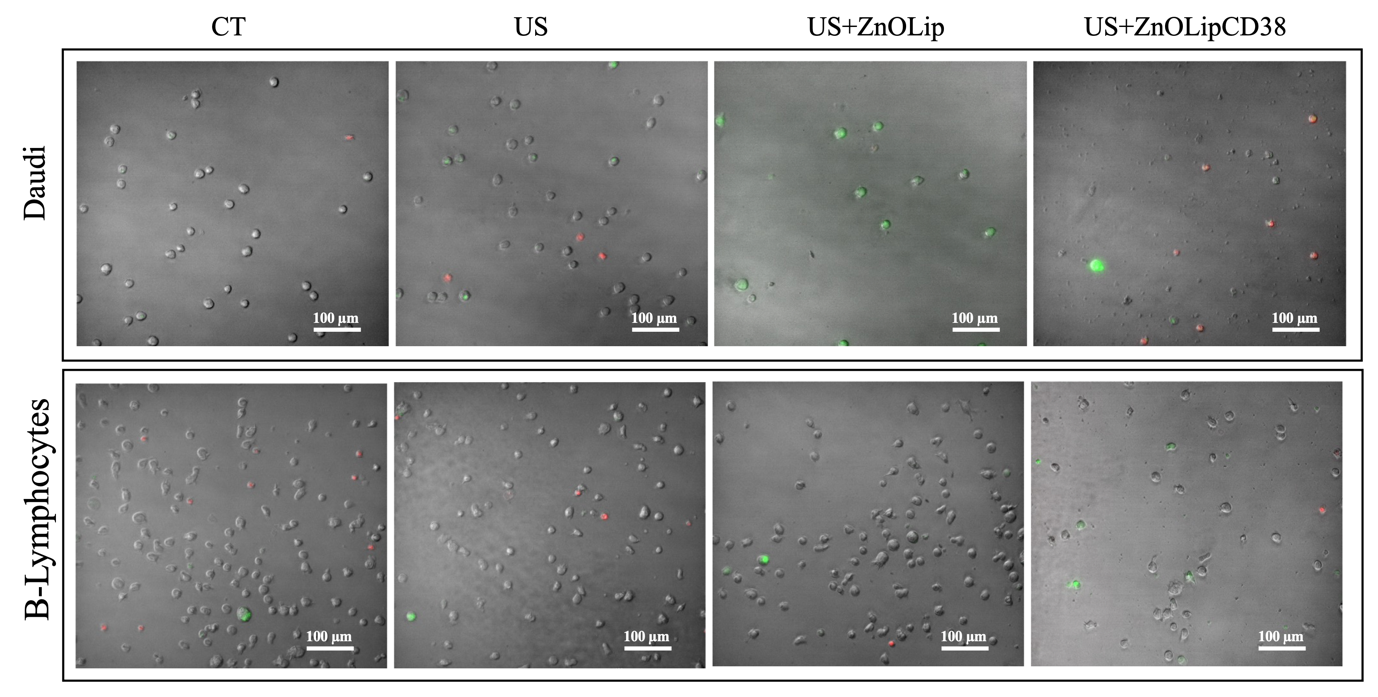
**

**Figure SI-4.** Cells membrane integrity was evaluated with PI dye (red), while Zn^2+^ presence inside cell was measured by Fluozin dye (green).

Figure SI-4 is a representative example of the images analysed to obtain the data shown in Figure 10, where for each experimental group 5 images were used to count how many cells were positive to PI and separately how many living cells were positive to FluoZin.
